# The view of synthetic biology in the field of ethics: a thematic systematic review

**DOI:** 10.3389/fbioe.2024.1397796

**Published:** 2024-05-28

**Authors:** Ayşe Kurtoğlu, Abdullah Yıldız, Berna Arda

**Affiliations:** Department of Medical History and Ethics, Ankara University School of Medicine, Ankara, Türkiye

**Keywords:** synthetic biology, ethics, bioethics, systematic review, technology ethics, responsible research and innovation

## Abstract

Synthetic biology is designing and creating biological tools and systems for useful purposes. It uses knowledge from biology, such as biotechnology, molecular biology, biophysics, biochemistry, bioinformatics, and other disciplines, such as engineering, mathematics, computer science, and electrical engineering. It is recognized as both a branch of science and technology. The scope of synthetic biology ranges from modifying existing organisms to gain new properties to creating a living organism from non-living components. Synthetic biology has many applications in important fields such as energy, chemistry, medicine, environment, agriculture, national security, and nanotechnology. The development of synthetic biology also raises ethical and social debates. This article aims to identify the place of ethics in synthetic biology. In this context, the theoretical ethical debates on synthetic biology from the 2000s to 2020, when the development of synthetic biology was relatively faster, were analyzed using the systematic review method. Based on the results of the analysis, the main ethical problems related to the field, problems that are likely to arise, and suggestions for solutions to these problems are included. The data collection phase of the study included a literature review conducted according to protocols, including planning, screening, selection and evaluation. The analysis and synthesis process was carried out in the next stage, and the main themes related to synthetic biology and ethics were identified. Searches were conducted in Web of Science, Scopus, PhilPapers and MEDLINE databases. Theoretical research articles and reviews published in peer-reviewed journals until the end of 2020 were included in the study. The language of publications was English. According to preliminary data, 1,453 publications were retrieved from the four databases. Considering the inclusion and exclusion criteria, 58 publications were analyzed in the study. Ethical debates on synthetic biology have been conducted on various issues. In this context, the ethical debates in this article were examined under five themes: the moral status of synthetic biology products, synthetic biology and the meaning of life, synthetic biology and metaphors, synthetic biology and knowledge, and expectations, concerns, and problem solving: risk *versus* caution.

## 1 Introduction

Synthetic biology is a branch of science that combines knowledge from biological fields such as molecular biology, biophysics, biochemistry, medicine, biomedical engineering, biotechnology, bioinformatics, and systems biology with disciplines such as engineering, physics, chemistry, mathematics, computer science, electrical engineering, and mechanical engineering (McDaniel and Weiss, 2005; Lam et al., 2009; Cheng and Lu, 2012; Keshava et al., 2018). This field studies the feasibility of creating organisms or parts using living and non-living materials. As a branch of engineering, synthetic biology endeavors to design and produce these organisms and their parts, utilizing these materials (Häyry, 2017).

Synthetic, meaning manufactured, often contrasts with natural. However, in the context of biology, it refers to the combination of two or more biological parts, either by design or by natural processes. Synthetic biology involves designing and creating biological tools and systems using engineering principles. While this definition has similarities with modern biotechnology, synthetic biology is distinguished by its emphasis on terms such as design, creation, construction, device, and system (Porcar and Peretó, 2014).

Synthetic biology is expected to have broader applicability than biotechnology in molecular biology and engineering. While biotechnology mainly focuses on using controlled biological circuits to design and produce new products, synthetic biology opens new avenues. It provides new opportunities to use artificial biological circuits to understand fundamental biological problems (Organisation for Economic Cooperation and Development, 2014).

The successful synthetic design of *Mycoplasma mycoides* has turned theoretical concepts into reality, demonstrating that a newly engineered life form or cell can survive and reproduce (Gibson et al., 2010). This achievement is particularly intriguing because, despite its synthetic origin, the cell is still governed by its genes, which are also synthetic. In essence, a synthetic cell has been brought to life, representing the creation of a new species. Such a groundbreaking scientific development inevitably requires ethical evaluation, given the potential positive and negative implications for the future.

Synthetic biology represents one of the most significant recent examples of the necessity for ethical considerations in the face of the unexpected becoming expected or possible. It has prompted the emergence of several crucial questions across a range of contexts. On the one hand, there are questions regarding the extent of responsibility researchers should assume and the degree of freedom they should enjoy in their work. Additionally, it prompts us to consider the limits of open science and how to protect scientific publications and results. Furthermore, the government’s stance on the public interest and the industry’s role in these discussions must be considered. Finally, the challenge lies in balancing the dilemma of benefit and harm. Given the state of bioethics in the modern era and its close relationship with technology, synthetic biology may represent a new turning point in bioethics (Kaebnick and Murray, 2013).

Ethics, especially bioethics, must be highly responsive to advances in their respective fields. However, due to the dynamic nature of the field, there are inevitably gaps in how ethics responds to these issues. Although gaps between practice and ethics are inevitable, the size of these gaps often paves the way for the emergence of undesirable problems. This leads to distrust of scientific disciplines and research or those conducting them. It is important to take a proactive approach to ethics and reduce the gap as much as possible (Askland, 2011). In order to reduce this gap, ethical awareness on the subject and the establishment of codes, especially for practice, should be established. For this purpose, it is essential to raise the awareness of the scientific world and society on ethical issues (Rappert, 2011).

This study aims to trace the emergence of ethics in synthetic biology over time. In this context, we systematically analyzed the theoretical ethical debates on synthetic biology from the 2000s to 2020, which coincides with a relatively faster and newer period in the development of synthetic biology. This research aims to present and discuss the basic framework of the theoretical debates on the ethics of synthetic biology for both medical and life scientists and ethicists.

## 2 Materials and methods

The study’s main question is defined as, *“What are the main ethical debates in the field of synthetic biology, and what are the contents of these ethical debates?”* The publications that met the criteria selected by the systematic review method were included in the analysis, and the relationship between synthetic biology and ethics was examined inductively. Based on the results, the main ethical issues related to the field, the problems that are likely to arise, and suggestions for solutions to these problems are given.

The data collection phase of our research involved a systematic literature review that included planning, screening, selection, and evaluation, all of which were conducted according to established protocols. We then undertook an analysis and synthesis process to identify key themes related to synthetic biology and ethics. We used the Preferred Reporting Items for Systematic Reviews and Meta-Analyses (PRISMA) checklist for the screening and analysis phases. However, due to the specific nature of our study, not all items on the checklist were applicable (Moher et al., 2009).

A literature search was conducted after determining the scope of the search and keywords. Databases were selected to provide search results in medicine, biotechnology, engineering, law, ethics, and bioethics. The literature search was conducted in the Web of Science, Scopus, MEDLINE Complete, and PhilPapers databases. Three of these databases were included because they are relatively the most comprehensive databases to provide an overview of the debate. PhilPapers was used to include the philosophical aspect of the scientific debate.

Because the field is relatively new and has a wide range of applications, the range of keywords was kept broad. For keywords related to synthetic biology, various bibliometric studies on the topic were used (Oldham et al., 2012; Hu and Rousseau, 2015; Raimbault et al., 2016; Shapira et al., 2017). In this context, keywords were defined as *synthetic biology*, *synthetic genomics*, *synthetic genome*, *synthetic genomes*, *synthetic gene*, *ethic*, *ethics*, *ethical*, *bioethic*, *bioethics*, *bioethical*, and *moral*. The search plans for keywords and databases are shown in Table 1.

**TABLE 1:** Search plan by database.

Database	Keywords
Web of Science	TS=(synthetic biolog* OR synthetic gen*) AND (ethic* OR bioethic* OR moral*)
Scopus	TITLE-ABS-KEY (“synthetic biolog*” OR “synthetic gen*“) AND (ethic* OR bioethic* OR moral*)
MEDLINE Complete	(synthetic biolog* OR synthetic gen*) AND (ethic* OR bioethic* OR moral*)
PhilPapers	(ethic* bioethic* moral*) (synthetic) (biolog*) *Fuzzy filter advanced*

TS = Topic; TITLE-ABS-KEY = Title, abstract, keywords

**FIGURE 1:**
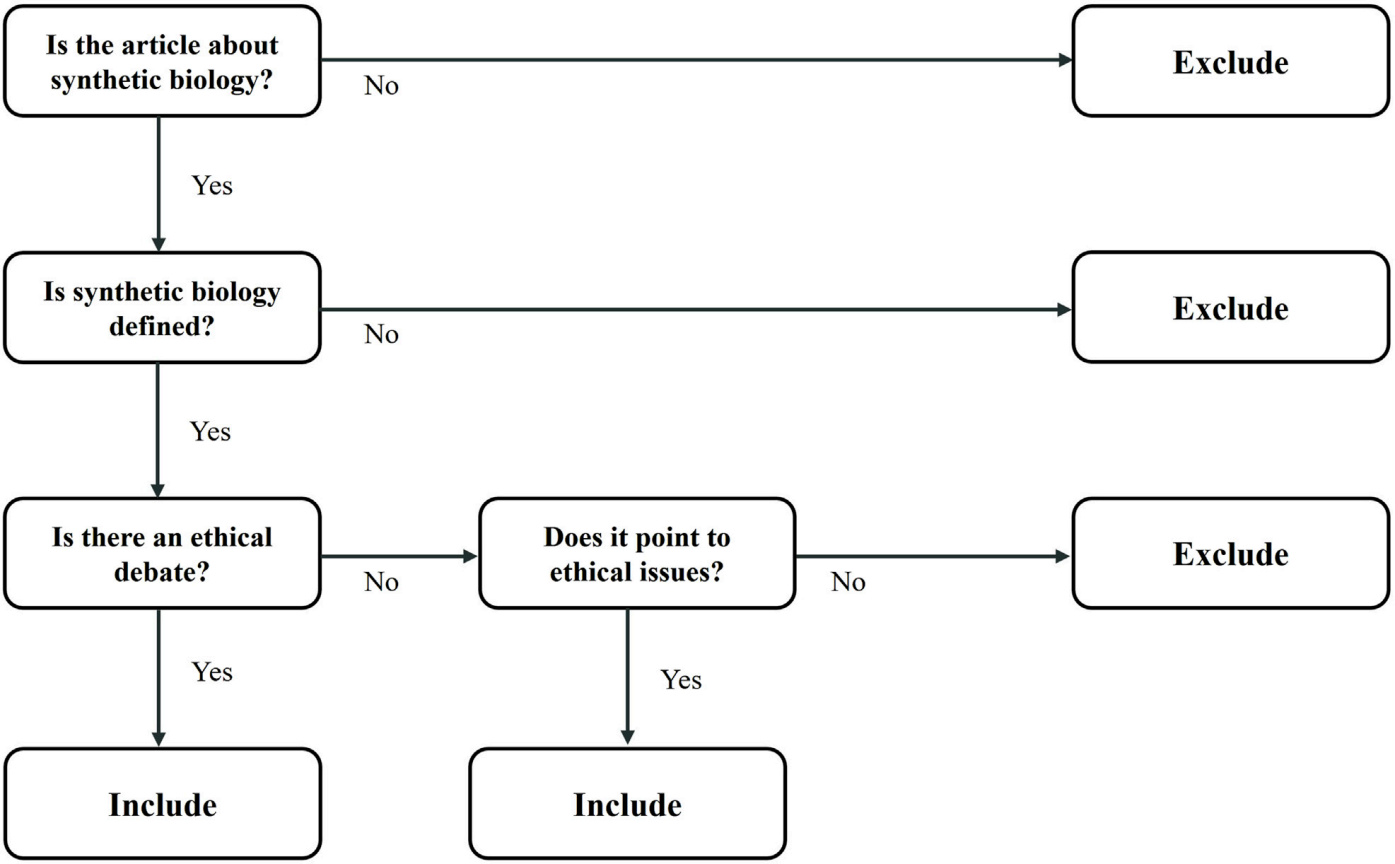
Inclusion decision diagram.

All searches were conducted between January and June 2021. Theoretical articles and reviews published in peer-reviewed journals by the end of 2020 were included in the study. There were no publication date restrictions. Many publications, including books, book chapters, editorials, commentaries, perspectives, web, and newspaper articles, were excluded. Empirical studies were also excluded. English language was used in the screening process. A decision tree was created to provide a relatively objective assessment of the evaluation by research question regarding the inclusion or exclusion of publications (Figure 1).

First, duplicates were removed from the publications obtained from the database search. The titles and abstracts were the screening content and keyword criteria. The next step was to read the full text of the publications selected for analysis. To avoid potential bias, the second author (AY) independently carried out a significant part of these steps, followed by a comparative discussion. The study excluded publications that mentioned ethics or bioethics in their keywords but did not include an ethical debate and those that did not have a definition of synthetic biology.

Thematic analysis was used to analyze the data and present the findings. Thematic analysis is often used to analyze systematic reviews and synthesize data, particularly in qualitative research. It involves reading and coding the texts and identifying descriptive analytical themes (Thomas and Harden, 2008). Thematic analysis allows for evaluation by revealing meaningful patterns in the whole data. With this method, the researcher defines and interprets the data, making it presentable to the reader. In this way, the reader can become familiar with extensive data-based research’s understandable and important aspects (Morgan, 2018). In this regard, the data was organized into five main themes. The quantitative analysis was mainly used descriptively to reveal synthetic biology and ethics’ quantitative course and main characteristics.

### 2.1 Limitations of the study

As it is quite difficult to carry out a quality assessment in the field of ethics, no such assessment was carried out in this study. However, an attempt was made to overcome this by ensuring the researchers were experts in ethics, philosophy, and bioethics. Again, it was always on the agenda of the researchers to approach the research with a reflexive attitude during the research (Dixon-Woods et al., 2006; Braun and Clarke, 2019).

As the search is mainly limited to publications in English, important studies in other languages may have been overlooked. Similarly, although the choice of terminology is quite broad, there may be new and different terminology uses. Nevertheless, a careful analysis has been carried out as far as possible. This was done by following the systematics of the retrieved publications.

It is also worth noting that Reviewer 2’s valid criticism that the role of biologists in ethical debates may not have been sufficiently covered and the question of what the authors think about this issue are topics that should be explained in the limitation section. In this respect, the fact that synthetic biologists working directly in the field are an important stakeholder will make their undeniable contributions and thoughts important. It is possible that their discourse may not have been sufficiently understood. This can be explained by the fact that criteria such as keywords that enable systematic review and the existence of ethical debate inevitably lead to the prominence of ethicists’ discourse. In this respect, experiential studies with biologists working in the field will be especially valuable in this context, as they will provide insight into the ethical implications of the research.

## 3 Results

### 3.1 Quantitative findings

The total number of publications obtained from four databases is 1,453. In the first step, duplicates were removed, and the number of publications was reduced to 888. The remaining 888 publications were screened for publication type, title, and abstract according to the inclusion and exclusion criteria. At this stage, 384 publications were excluded because they did not meet the publication type criterion (peer-reviewed article or peer-reviewed review), and 255 were excluded because they were unrelated to the research topic.

**FIGURE 2:**
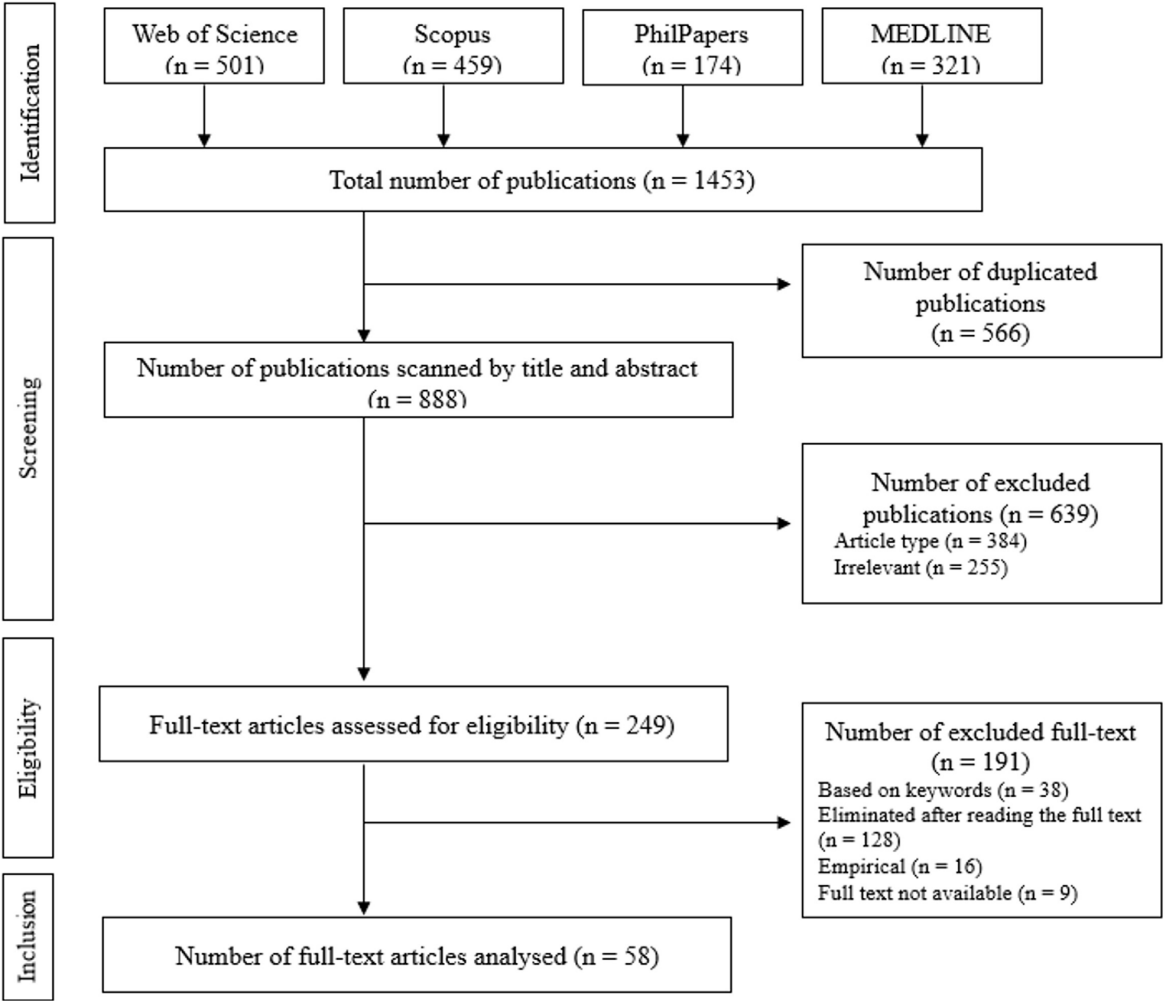
Flowchart of selection and inclusion of publications.

**FIGURE 3:**
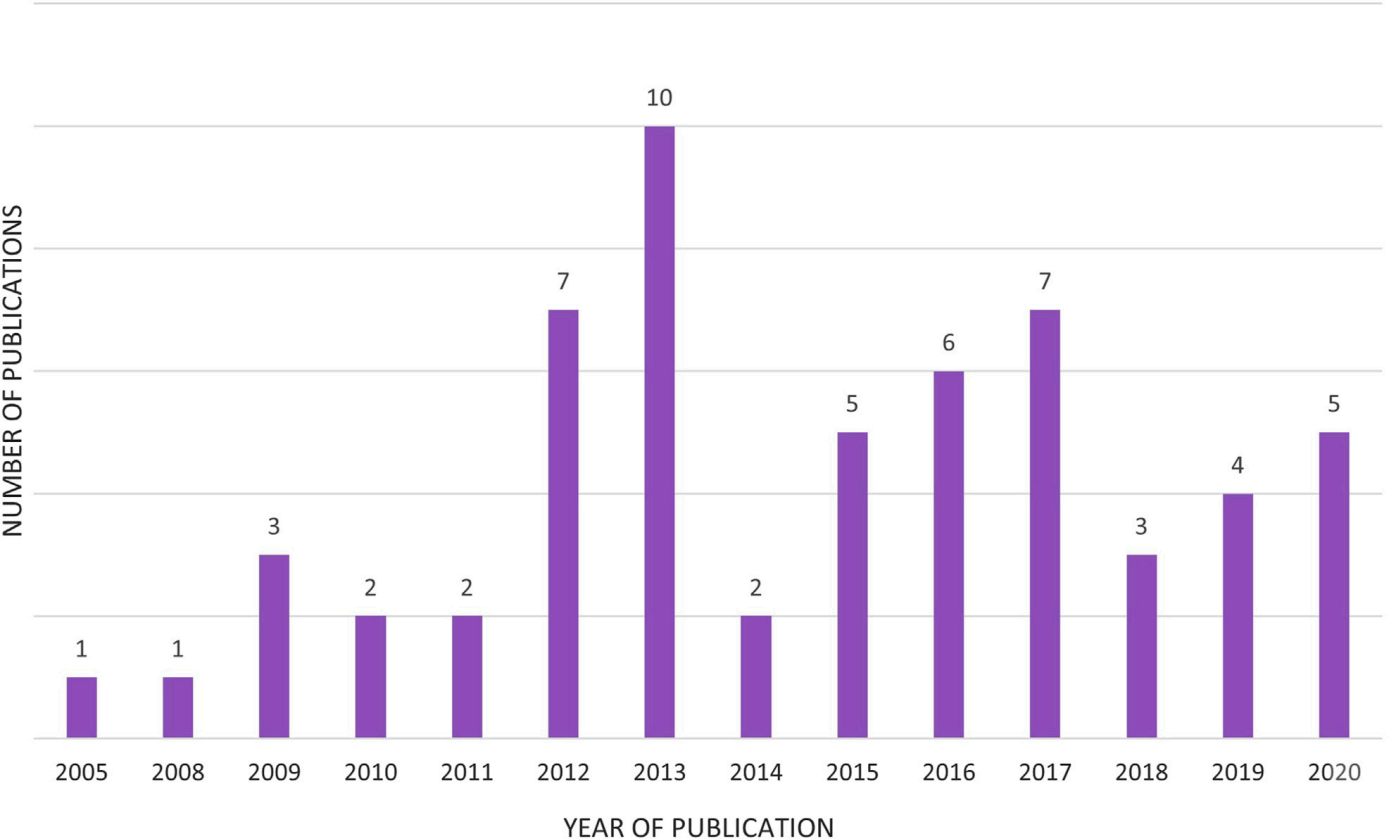
Number of articles in sample (n = 58) sorted by year of publication.

The full texts were read (n = 249), and the selected publications were analyzed using the inclusion and exclusion criteria and the decision tree diagram. During the full-text reading, keyword and title elimination was performed again, and publications (n = 38) that did not contain synthetic biology* and ethic*/bioethic* in their keywords and/or titles were excluded (Figure 2). Accordingly, 191 of the 249 publications were excluded from the full-text reading because they needed to meet the criteria; 58 publications between 2005 and 2020 were included. Figure 3 shows the distribution of included publications by year.

### 3.2 Thematic analysis

Detailed analyses of the publications are presented in Supplementary Material S1. Articles were screened for prominent ethical debates related to synthetic biology and conclusions and recommendations, if any. The researchers thematized the data to create similar and meaningful content (Table 2).

**TABLE 2:** Thematic analysis of the ethical and conceptual dimensions of synthetic biology.

Theme	Description	Key questions	Implications	Main concepts
The Moral Status of Synthetic Biology Products	Ethical examination of created life through synthetic means, focusing on intrinsic and instrumental values	What moral obligations do we have to synthetic life forms?How do we balance innovation and ethics?	Challenges traditional views of life and demands a reevaluation of ethical frameworks	Intrinsic value Instrumental value
Synthetic Biology and the Meaning of Life	Discusses how synthetic biology is changing our understanding of the nature of life	Does the value or meaning of life change when it is created synthetically?	Prompts a rethinking of the philosophical definition of life	Meaning of life
				Value of life
				Natural-artificial distinction
				Viewing synthetic biology as an activity of creating technological innovation
				Changes in the evolutionary process
Synthetic Biologyand Metaphors	Examines the role of metaphors such as “playing God” in the ethical discourse of synthetic biology	How do metaphors shape our perceptions of the ethical implications of synthetic biology?	Metaphors can both clarify and complicate ethical debates	Machine metaphor
				Playing God
Synthetic Biology and Knowledge	Focuses on the acquisition and implications of synthetic biology knowledge	What responsibilities come with new knowledge? How should it be managed?	Issues of control and dissemination of potentially dangerous knowledge	Dissemination of knowledge
				The meaning and ethical implications of the knowledge produced
				Ethics of knowledge
				Dual-use
				Intellectual property
Expectations, Concerns, and Problem Solving: Risk vs. Caution	Balances the potential benefits of synthetic biology with ethical and safety concerns	How do we manage the risks while pursuing the benefits of synthetic biology?	The need for regulations and ethical guidelines to manage risk	Potential benefits and harms of synthetic biology
				Utilitarianism
				Benefit sharing
				Consequentialism
				Moral obligation
				Proactive approach
				Precautionary approach
				Biosafety
				Biosecurity
				Bioterrorism
				Commercialization
				Synthetic biology tourism
				Risk assessment
				Physical harm
				Non-physical harm
				Professional ethics
				Practical ethics
				Deciding in the dark
				Safe-by-design
				Shared responsibility
				Conservation ethics
				Good engineering practice
				Ethical evaluation in technology
				Governance measures
				Deliberative approach
				Social awareness
				Fair distribution

The content that emerged from the screening and analysis based on the research question was reflected in five different themes: 1) the moral status of synthetic biology products; 2) synthetic biology and the meaning of life; 3) synthetic biology and metaphors; 4) synthetic biology and knowledge; 5) expectations, concerns and problem solving: risk *versus* caution.

#### 3.2.1 The moral status of synthetic biology products

The main issue that gives rise to this theme is views on the moral value of synthetic biological products. In particular, the concepts of intrinsic and instrumental values underlie the discussions on the moral position of the synthetic biological product. In this context, a utilitarian approach is evident, determined by the orientation towards human health and wellbeing as instrumental. However, there is also the view, especially about the later theme, that the synthetic biological product can also be attributed an intrinsic value, which is possible with its existence in the field of existence. It is possible to say that the content that stands out in this theme is closely related to the theme of synthetic biology and the meaning of life.

#### 3.2.2 Synthetic biology and the meaning of life

What gives rise to this theme are the concepts and debates about the place of the synthetic biological product within the existing sphere. In this context, a conceptual framework emerges in which the meaning of life, the value of life, the distinction between natural and artificial, the view of synthetic biology as an activity of creating innovation, and the transformation of the evolutionary process come to the fore. The question of how the product of synthetic biology can be defined and how it can be positioned in the field of existence is problematized, especially in the debates about artificial and natural existence. The view of the synthetic biology product as a product of the act of creation as a technological product has also emerged as an important evaluation area. The fact that it points to a revolutionary point in the evolutionary process by demonstrating the feasibility of products in synthetic biology is also among the prominent discussions. In general, the relationship of all these discussions and contents to the meaning and value of contemporary life seems obvious.

#### 3.2.3 Synthetic biology and metaphors

It was observed that metaphors are occasionally used in ethical discussions about synthetic biology and that these metaphors are used as arguments. In this respect, it can be said that the machine metaphor and the playing God metaphors come to the fore. These two contents have important references in terms of value. It can be said that the metaphor of playing God is used with the ontological meaning attributed to life, and a critical attitude is developed through it. On the other hand, the machine metaphor stands out in relation to the current perception of technology and its functional dimension. However, the ontological aspect of this discourse is also important in terms of the moral position to be attributed or ascribed to the synthetic biology product.

#### 3.2.4 Synthetic biology and knowledge

The knowledge produced in synthetic biology is an essential category of discussion. The circulation and distribution of knowledge is an important topic of interest and discussion. Notably, some authors emphasize the need for an ethics of knowledge. About this theme in addition to discussing information in terms of risk, such as the dual-use of information, issues, such as the value of information as a commodity, are also discussed. Access to produced knowledge and commercialization of knowledge are on the agenda as essential sources of interest and concern.

#### 3.2.5 Expectations, concerns and problem solving in synthetic biology: risk *versus* caution

This theme can be considered the most substantive. In particular, synthetic biology’s current and potential benefits and risks and harms that may arise despite these benefits have a very crucial place in synthetic biology ethics. A large number of concepts are related to these concerns. Naturally, the content that makes up this theme is spread over a broad spectrum. One peculiarity that makes this theme special is that many suggestions related to technology ethics are given in relation to the categories of risk and problem. In this respect, the prominent concepts are risk assessment, proactive approach, safe-by-design, etc., which can be seen as related to prudence. Again, the emphasis on deliberative and shared approaches in democratic societies to anticipate and solve possible problem clusters is evident.

## 4 Discussion

Although an utterly artificial cell has not yet been created, creating a synthetic genome and its insertion into the cell has been successfully achieved (Melin, 2021). Such possibilities, which can be considered one of the most critical scientific steps in the history of humankind, have ethical and social consequences, and discussions on these issues are inevitable. In this respect, it is noteworthy that the number of publications addressing the ethical dimension of synthetic biology (regardless of the publication type) has increased, especially since 2011. This situation is in line with the demonstration of the feasibility of the field by J. Craig Venter in 2010 (Gibson et al., 2010). Furthermore, if we look at the topics of the papers, we see that the more recent ones are more oriented toward practical solutions.

In contrast, the earlier ones focus more on theoretical and philosophical studies. Empirical studies have also gained weight in more recent periods; this situation parallels the development of the field and the more visible application areas. This situation may also be related to the importance of producing ethical discourses against possible damages or risks that occur or may occur during practical applications. Most of the publications are of Western origin and written by researchers working in very different disciplines related to synthetic biology. Theological discussions on synthetic biology were also observed.

No directly negative attitude or opinion was found in the discussions about the feasibility of synthetic biology in the publications. The discussions were spread over a spectrum, with a predominance of positive expectations based on utilitarianism regarding the feasibility of synthetic biology and the need to take various measures to balance them (basically positive, in our opinion). In short, it is difficult to speak of the existence of an approach that finds synthetic biology studies ethically unjustifiable. The trend change is that synthetic biology’s feasibility has become apparent over time. The emergence of ethical debates’ theoretical and practical aspects may lead to a reflective equilibrium. In this context, ethical discussions made practices feasible on the one hand and created optimism about their verifiability on the other.

### 4.1 The moral status of synthetic biology products

In the debate over the moral status of synthetic biology products or synthetic organisms, intrinsic and instrumental or extrinsic values are prominent concepts. The distinction between intrinsic and instrumental value has a long history in the environmental ethics literature (Bedau and Larson, 2013; Preston, 2013). This distinction distinguishes between values found in nature that serve a human need or purpose and those considered to exist in nature independent of any human desire or interest (Preston, 2013).

The intrinsic value of nonhuman beings and the normative position of the natural *versus* the artificial are significant for discussing morality in synthetic biology. One of the fundamental questions raised by synthetic biology is whether organisms’ synthesis is compatible with nature’s intrinsic value (Bedau and Larson, 2013).

Synthetic biology enables the design and construction of synthetic life forms in a deliberate, conscious, and rational manner, with unprecedented flexibility and precision of control. In this context, the construction process by which synthetic life is created may be quite different from the natural evolutionary process that produces known natural life forms (Bedau and Larson, 2013).

One view is that one should ask how synthetic biology can conflict with the so-called intrinsic value of life (Link, 2013). In this context, to morally question synthetic biology, one should first focus on the intrinsic value of life and the relationship of synthetic biology to these values. Since synthetic biological products gain meaning in nature by coming into existence, it takes work to consider them valuable. In this case, the only way to make sense of the appeal to the intrinsic value of existing life would be to remove its close connection to protection from harm.

According to another view, almost anything can have an instrumental or extrinsic value. Indeed, instrumental value is the reason for the existence of synthetic organisms. However, once a synthetic organism is created, it exists for itself (with purposive behavior), even though humans determine its ends. In other words, although synthetic biologists define their ultimate interests, these beings also have their proximate interests. Through their proximate interests, these entities also have instrumental value and, thus, intrinsic value. In this context, what is important in defining intrinsic value is not whether an entity is natural or artificial but whether it is a living being (Coyne, 2020). In this case, the synthetic entity may have intrinsic value because it is instrumentally valuable.

Synthetic biology and its products have many useful purposes. From this perspective, the product of synthetic biology has instrumental value. However, it is worth discussing whether synthetic organisms also have intrinsic value, which provides reasons to create them independently of the benefits they can provide humans.

Morally, the fact that an entity has intrinsic value also affects moral judgments about it; it requires positioning it as different from a simple object in terms of morality without attributing any external value to it. This question of moral status is closely related to the discourses on synthetic biology and the meaning of life. What a synthetic biological product is in terms of life inevitably affects its value. In this context, the meaning of life points to an ontological position that also determines its moral status and value. This situation shows that the link between ontology-epistemology and ethics established in discussions of ethics in general is also valid for this research.

### 4.2 Synthetic biology and the meaning of life

With scientific advances and innovations in biology and the life sciences, disagreements, and debates continue about what should and should not be included in the definition of life and the nature of definitions (Mariscal, 2021). Despite these difficulties, both philosophers and scientists have attempted to define life. The reason for this interest in defining life is the new sciences and technologies, such as artificial life, synthetic biology, etc., which challenge some of the traditional characteristics associated with life and further complicate the problems of life (Mariscal, 2021). For synthetic biology, which has significantly changed established ideas about life through creating life, the meaning of life and its ethical implications are of fundamental interest.

In discussions of the meaning of life, there is a particular emphasis on an ontological difference between humans and other beings, including the product of synthetic biology. This also leads to an important distinction in terms of the moral position of beings and is associated with a fundamentally anthropocentric attribution of morality (Gómez-Tatay et al., 2016). This is in line with the interpretation presented in the previous theme, according to which the ontological orientation regarding the meaning of life affects morality.

In some of the publications included in the analysis, it was emphasized that researchers should use the term life through a very detailed analysis (Funk et al., 2019). In this context, there are concerns about using the concept of life. In synthetic biology, attention is drawn to a positive and developmental discourse on the field, avoiding the restrictive use of life. In this respect, presenting the concept of life in a controllable form that provides a consensus for researchers is an important requirement. It is emphasized that an arbitrary and inappropriate use of the concept of life can lead to ethical problems. Nevertheless, attention is drawn to the mutability of the concept of life with developments (Funk et al., 2019).

The distinction between natural and artificial is essential for the definition of life. It is believed that what is being produced, especially in synthetic biology, has significantly changed or will change the difference between natural and artificial (Gómez-Tatay et al., 2016). While the things produced by humans until synthetic biology were generally associated with artificiality, it can be said that the positioning of the synthetic biology product as an organism blurs this difference. While synthetic biology products contain artificiality in terms of being designed, they also contain naturalness in terms of being an organism and having vitality. Ethically, this is a controversial issue, with the possibility of being associated with instrumentalism in terms of artificiality and intrinsic values in terms of naturalness.

For some authors, the distinction between natural and artificial creation is the most fundamental ethical debate. In this respect, the argument of playing God, also discussed in the theme of metaphors, comes to the fore. Although it is stated that an analysis should be made by returning to the created product itself from an ethical point of view, it is noted that the act of creating human beings with synthetic biology is criticized, at least rhetorically, with the argument of playing God. While the artificial overcomes the natural, it is criticized that humans can influence the natural process of life and evolution by committing a very arrogant act with the act of creation (Heyd, 2012).

It was mentioned that the synthetic biology product contains contradictions in referring to fabrication by characterizing it as a technological product and talking about an act of creation. Mechanistic production based on fabrication involves prediction and control. However, the widespread use of this discourse in synthetic biology will cause some fundamental problems. These include an unnecessary self-confidence that organisms can be created reliably, a closure to external evaluation due to the emphasis on doing things right internally, and self-criticism regarding the value of what is produced (Boldt, 2013). These evaluations concern production processes, product prediction, and limiting problems, especially in synthetic biology. These concerns are addressed in the theme expectations, concerns and problem solving in synthetic biology: risk *versus* caution.

It has been mentioned that the creation process in synthetic biology represents a break with the current state of nature in terms of the evolutionary process. This means the newly produced biological product differs from the continuity product from generation to generation. This break in continuity is seen as an area of concern. At the center of this concern is the impact on normative rules that are important in the evolutionary process, especially regarding environmental ethics. The current evolutionary process and the continuity of the natural world are an important basis of normative criteria for classical environmental ethics. However, synthetic biology may cause an interruption and intervention in the evolutionary process in this sense, making it impossible to establish norms and rules. This will be an important problem, especially in the context of environmental ethics (Preston, 2008). In bioethics, the development of new technologies has historically been crucial to developing the field and establishing ethical norms. However, it is also an important issue that synthetic biology, with its unique characteristics, is generating new debates by affecting the structure of the natural situation and the previous environment, and it will take more work to set limits to norms.

Considering the current developments and the dynamic structure of synthetic biology, although it is challenging to define life, any definition of life or discourse about it will profoundly affect ethics with its ontological assumptions and will be decisive for the ethical position towards these beings. Whether these forms are accepted as life itself or treated as purely instrumental will also determine the ethical position toward them. Regarding the problems of foresight and the boundary between artificiality and naturalness in terms of creation, the act of creation also has a meaning related to problems of risk and the future.

### 4.3 Synthetic biology and metaphors

Some philosophers and linguists consider metaphors essential for understanding politics, social life, and human thought (Groeger, 2010). The recent use of metaphors in the life sciences and their historical transformations have attracted the attention of social scientists, philosophers, and scientists (McLeod and Nerlich, 2017). It is stated that metaphors are not only linguistic expressions that beautify language but also influence human thoughts and actions about the world (Lakoff and Johnson, 2015; McLeod and Nerlich, 2017). Words or discourses also have consequences that can affect ethics, social life, human actions, and the economy (McLeod and Nerlich, 2017).

Linguistic discourses are particularly affected by scientific developments in biology and synthetic biology. However, it also affects the social sciences in terms of expectations, anxieties, and the meaning given to life (McLeod and Nerlich, 2017). Initially, it was noted that metaphorical discourses related to synthetic biology were associated with greater scientific activity and power. Later, it was found that this discourse was balanced by metaphors pointing to responsibility (Check, 2006). Overcoming trust problems in science and the concerns of society are crucial to these discourses. In addition, it is emphasized that language should be used carefully and responsibly in the scientific field of metaphors (McLeod and Nerlich, 2017).

Concerning emerging technologies such as synthetic biology, metaphors are among the elements that should not be overlooked. This is because these discourses also contain content related to paradigm shifts and society’s reactions to them (Falkner, 2016). Synthetic biology also stands out as a field in which metaphorical discourses are produced. Metaphors such as playing God and creating life are predominantly used, while function-oriented discourses such as living machine are also added (Matern et al., 2016). Although metaphors do not emerge as a dominant concept in the texts we analyzed, it can still be said that meaningful content has emerged.

Synthetic biology provides an example of how scientists use the machine metaphor to organize their thinking about a system (Holm, 2020). The machine metaphor used in synthetic biology is recognized as a powerful conceptual expression. The machine metaphor is closely related to the ontology of the synthetic biology product. Suppose it is sufficient to know the structure of the simple molecules in that structure to explain complex molecules (assuming that there are laws and regularities governing the behavior of simple parts). In that case, it is sufficient to identify and analyze the genetic structure of an organism to explain its function. According to this view, if the parts and functions of an object are known as a relation, the overall behavior of that object can be reliably predicted (Boldt, 2018).

There is a close relationship between metaphysical discourses on synthetic biology and the moral status of organisms. If an intrinsic value is attributed to the product of synthetic biology, then the use of these organisms for human beings can be justified. If a machine analogy is made, an instrumental value is questioned. Therefore, referring to synthetic biology products as machines or organisms has a different meaning than a simple metaphorical discourse (Deplazes and Huppenbauer, 2009). The distinction between living beings and machines is crucial in determining their moral positions. For example, machines, such as computers, have no intrinsic value; they are valuable only insofar as they can be used to achieve valuable ends. Machines also lack interests and rights. Organisms, on the other hand, have all of these. It has been argued that the erosion of meaning ascribed to the distinction between organisms and machines may lead to organisms being wrongly equated with a lower moral status than they have (Douglas and Savulescu, 2010).

The meanings of machine and organism concepts are an important topic of discussion, recalling the previous theme. A predictable situation can be pointed out in the machine metaphor by defining the synthetic biology product as a machine. However, defining this product as an organism would inevitably limit the possibilities of this foresight. This metaphorical use will create a meaningful difference in ethical approach, at least concerning the problem of risk and foresight.

The playing God argument is also an essential ethical metaphor. Playing God describes morally unacceptable actions and implies that some absolute moral boundaries have been crossed. This argument embodies the idea that there are possibilities that should not be realized and choices that should not be made. It also expresses concerns about the arrogance of tampering with sacred things (Grey, 2012). The argument for playing God has distinctly theological elements. In addition, secular use of the argument is every day today. According to this usage, nature plays the role of God, and some things should be left to nature. When humans try to do the right thing in nature, they go against nature (Weckert, 2016). In secular terms, the argument for playing God is interpreted as going beyond being omnipotent, omniscient, and benevolent and acting in ways that ignore established constraints on human knowledge, power, and benevolence (Coady, 2009).

When people are concerned about implementing the latest scientific and technological discoveries, they express this concern regarding playing God. The concern is that these practices may involve an unwarranted reliance on knowledge, power, and virtue beyond what is reasonably permissible for human beings (Coady, 2009).

Playing God addresses three religious concerns regarding synthetic biology debates: 1) A deontological method expresses that certain boundaries should not be violated. This boundary is considered God’s domain and off-limits to human action. 2) Playing God may express judgments about certain attitudes, values, or virtues that appropriately respond to God’s nature, including awe, wonder, and humility, with respect and reverence corresponding to natural boundaries. 3) Playing God means proposing simple, even isomorphic correspondences between the world’s order, defined by concepts such as fixed kinds or, more broadly, natural kinds and God’s creative will and purposes (Lustig, 2013).

One of the main ethical criticisms of the playing God argument is the danger that it leaves the underlying moral principle unstated so that it can function as a rhetorical device that renders moral debate meaningless rather than illuminating. Serious uses of this argument, therefore, require a moral framework or appeal to a moral principle, and the assumed principles should be clearly articulated to overcome ambiguity and avoid confusion (Grey, 2012).

### 4.4 Synthetic biology and knowledge

It is noted that synthetic biology is not only a practical but also a knowledge-generating field and, therefore, deserves special attention. In this context, it is emphasized that in synthetic biology, attention should be paid to the stages of knowledge production and the meaning and ethical significance of knowledge. Accordingly, ethical attention and care should be given to the processes of knowledge production. Those who produce knowledge should be interested in the future risks of the knowledge they produce. While states and knowledge producers have a role, ethicists should support knowledge producers by offering different perspectives. It is also important not to inhibit knowledge production (Douglas and Savulescu, 2010).

A balanced approach comes to the fore in the knowledge debate. On the one hand, there are utilitarian expectations from synthetic biology; on the other hand, it is seen that the category of knowledge itself can be considered a risk area. In particular, ethical attention to and care for knowledge includes concern about the misuse of knowledge. In this context, Douglas and Savulescu (2010) point out that the distribution of knowledge is a relatively minor area of discussion in bioethics; however, due to the critical role of synthetic biology in knowledge production as a life science, ethics of knowledge is inevitably needed in this field. However, it is appropriate to note that since the publication of Douglas and Savulescu’s (2010) article, access to information has been on the bioethics agenda under various headings. In this context, issues such as open access and dual-use have taken their place among the essential topics of bioethics, as they involve issues such as making information accessible to large masses while at the same time ensuring its safe use. Especially in synthetic biology, dual-use has become a critical ethical debate.

Detailed ethical analyses in the dual-use context of life sciences research are important to assess the research’s actual and potential benefits and risks. These analyses will also identify salient policy options, and each option will affect the relationships between present and future benefits and burdens and their recipients and bearers. The design and selection of these options will primarily involve the application of various ethical principles, including human rights principles (such as the right to life, freedom of expression, and academic freedom), the principle of utility, and justice (Miller and Selgelid, 2008).

Indeed, the issue of dual-use is a crucial area of discussion in the ethics of knowledge. Significant technological developments also involve serious risks, and the rapid developments in the life sciences have increased the importance of the dual-use phenomenon (Tucker, 2012). As a new technoscience, synthetic biology promises useful applications in many fields, but it also carries the risk of misuse (Kelle, 2012).

Dual-use in synthetic biology is mainly associated with biosafety issues (Newson, 2011; Newson, 2015; Rager-Zisman, 2012; Raho, 2014; Heavey, 2017; Holm, 2017). Synthetic biology offers significant opportunities with potential applications such as large-scale drug synthesis, biofuel production, green chemical production, and the development of bioremediation tools for antibiotic-resistant microorganisms. However, synthetic biology also has advanced technologies for producing organisms with the ideal characteristics of biological weapons, raising concerns that published scientific information could be used for malicious purposes. Creating a biological weapon, processing and developing agents, and isolating and disseminating them requires skill and equipment. Therefore, any medical advance that facilitates the development, use, or delivery of a process has the potential to be used for harmful purposes and is therefore considered dual-use. The scientific knowledge generated in the field of synthetic biology is growing cumulatively. Access to this growing body of scientific knowledge, markers, or equipment increases the risk of more dangerous biological weapons (Rager-Zisman, 2012).

In the context of synthetic biology, there are several suggestions to address dual-use concerns. The main point of these concerns is that scientists need more information about the consequences and risks of synthetic biology research. However, scientific developments will reveal the research’s benefits and harms (Heyd, 2012). At this point, scientists are responsible to society and should take responsibility for biosafety as part of their research, especially when conducting research with dual-use concerns (Heyd, 2012; Rager-Zisman, 2012). Ethical judgments should not be made in ignorance. In balancing potential harm against potential benefit, it has been emphasized in this research that care should be taken to minimize dangerous practices. It is an ethical imperative to make arrangements that enhance benefits and minimize the likelihood of worst-case scenarios (Heavey, 2017). A systematic precautionary governance approach has been proposed for the dual-use of synthetic biology. National and international cooperation is needed to determine the scope, content, and processes of this systematic and sustainable approach (Kelle, 2013).

Another issue related to the ethics of knowledge is based on concerns about the commercialization of the information produced and how it can be used for the public good. In this context, it has been argued that while protecting the public interest, regulations should be made on intellectual property rights related to information; it has also been stated that states should develop policies that help researchers and those who do not have easy access to information and should be regulators (Saukshmya and Chugh, 2010).

Discussions about equitable access to knowledge have also come to the fore. It can be said that a socialist perspective on access to and distribution of information is important for the common good. Rather than seeing knowledge production and knowledge as a value, it is crucial to position knowledge as something fundamental that concerns all people and societies today. This discourse is consistent with recent discourses suggesting that everyone should have equal access to the benefits of emerging technologies, including synthetic biology. It is also noteworthy that there is concern that inequality based on knowledge and technology, including knowledge, will further widen the gap between developed and developing countries.

### 4.5 Expectations, concerns and problem solving in synthetic biology: risk *versus* caution

One of the dominant issues in discussions about synthetic biology and ethics is the field’s risks and possible negative consequences. In this context, the concept of risk and different problems are mentioned. However, the status of being the last modern technology attributed to synthetic biology also distinguishes risk content from other technologies. In synthetic biology, risk involves an ontological perception of threats to humans, nature, and the world; in this sense, it differs significantly from mechanistic technologies. This situation has led to the emergence of earlier proactive and precautionary approaches. Some authors have criticized these approaches as an overly fearful reaction to synthetic biology research and its products. However, the benefits to humanity and the belief that synthetic biology products will solve current health, environmental, and economic problems make these discussions exciting and complex.

It is possible to see an interesting ethical discourse in which synthetic biology products and synthetic biology research have a meaning similar to Kant’s categorical imperative and where translational research and the practical implications of synthetic biology are positioned as a moral obligation to be demonstrated (Heidari Feidt et al., 2019). Such an analysis offers a different interpretation of scientific research, determined by a utilitarian approach. A utilitarian approach is expected to ethically incentivize these studies and benefit society and humanity more broadly (Heidari Feidt et al., 2019). At the same time, it is likely that this debate will not be confined to the academic environment but will be driven by political and economic expectations.

The unique characteristics of synthetic biology have positioned it as a field that promises opportunities but carries serious risks. This means that synthetic biology offers fundamentally unpredictable opportunities and applications. This is because synthetic biology products’ structures can evolve and change unpredictably (Holm, 2019).

Ethical concerns about synthetic biology fall into two main categories. Physical harms generally include concerns about the risks and consequences of synthetic biology regarding health, safety, and environmental impacts, while non-physical harms include concerns about moral values, ethical consequences, public welfare, and social justice (Newson, 2011; Race et al., 2012).

One of the most physical risks is the impact on living organisms and ecosystems. The release of synthetic biology-derived organisms, unique in nature, into the environment raises doubts about how these organisms may interact with the environment. Another risk is contamination, which could result from accidently releasing genetically engineered organisms into the environment. Unlike synthetically produced chemicals, which tend to have well-defined and predictable properties, biological organisms are more difficult to control. Any accident or release that is not managed correctly can lead to the crossing of these organisms with other organisms found in nature and uncontrolled reproduction, threatening biodiversity (Rohregger et al., 2020). Synthetic biology may also pose risks in the energy sector, which is expected to be the most widely used area. In particular, biomass as a fuel feedstock is expected to lead to the categorization of all of nature as biomass and to an exponential increase in the use of biomass on the planet. Potential risks in agriculture include uncontrolled environmental leakage, difficulty in controlling new or resistant pests, and damage from increased invasive species resistance (Rohregger et al., 2020).

When considering physical risks, biosafety and biosecurity are among the most frequently discussed issues (Newson, 2011). In the context of synthetic biology, the issue of biosafety arises from the intentional or unintentional release of synthetic organisms outside the laboratory. As a result of the release of such organisms, there is a risk of unexpected interactions with other living organisms. The fact that synthetic biologists are working with living organisms and modifying them at unknown levels may result in these organisms having complex structures that cannot be fully known or understood. This poses significant threats to agriculture, the environment, and human health (Newson, 2011; Raho, 2014). The biosafety debate also includes what is safe in synthetic biology and who should determine it. While there is consensus that there should be strong standards, there needs to be complete agreement on what they should be.

Synthetic biology offers the potential to create, replicate, and modify potentially dangerous viruses or bacteria. Biosecurity issues related to synthetic biology raise significant questions about the exposure of the general population to risk. These questions arise from the potential use of synthetic biology products by those who wish to commit malicious acts, such as bioterrorism (Newson, 2011; Raho, 2014). In this context, it is difficult to determine the level of potential harm and practically impossible to control the actions of individuals or groups (Ahteensuu, 2017). Most biosecurity concerns stem from the relative ease with which materials can be used to conduct synthetic biology research in settings other than a research center. This raises dual-use concerns, also discussed under the synthetic biology and knowledge theme.

Non-physical harms include goals or practices of synthetic biology that may harm the wellbeing of individuals and communities (Newson, 2011; Race et al., 2012), although the distinction between them is not very clear. Possible non-physical harms of synthetic biology include the role of humans in creating new beings and how this will affect self-concepts and relationships with the environment (Newson, 2011). The moral status discussed in the previous themes is also included in non-physical harm.

Fair allocation of synthetic biology knowledge, products, and commercial benefits are among the potential non-physical harms. The ability of synthetic biology to exploit the reproductive mechanisms of living organisms is an essential advantage for the field. This way, developing devices, parts, or processes produced in the laboratory may not be as costly as other emerging technologies. However, there are concerns about the infrastructure needed to make these technologies available for everyone’s benefit (Newson, 2011). Another potential non-physical harm that seems at odds with equitable distribution is commercial benefit. In synthetic biology, as in all scientific disciplines, there is a need to encourage innovation while being mindful of the constraints that intellectual property can impose. However, concerns have been expressed about confrontation with patent holders. In this context, it has been suggested that different levels of intellectual property protection should be provided for different synthetic entities, with more protection for more complex organisms (Newson, 2011).

Decision-making in risky situations is an essential area of debate in synthetic biology. Risk and decision-making have long been on the agenda in technology, economics, and management. However, as mentioned above, the differentiated technological nature of synthetic biology, while changing the dimension of risk, is also associated with the concept of *deciding in the dark*. This concept refers to the inherent difficulty of making decisions and predicting risks in this field (Holm, 2019). One approach referred to in this case is the precautionary principle.

The precautionary principle is a decision-making tool that helps when highly uncertain risks must be managed. Initially developed in environmental law and policy, its recognition increased after the 1970s due to the global, potentially irreversible, and largely unknown impacts of greenhouse gases and related climate change. Over time, the principle has been applied in areas such as health protection and the regulation of new biotechnologies, going beyond environmental issues; it has become an essential element of practical ethics as one of the effective principles in issues related to the protection of the environment and human health and related policies (Holm and Stokes, 2012). Synthetic biology is one of these fields.

In synthetic biology, the precautionary principle has been considered quite comprehensively, and it has been proposed to be used as a risk assessment approach that considers more than one factor. Recent studies advocate the involvement of society in the process as a requirement of a democratic society. Considering the future risks and the dynamic structure of synthetic biology, this discourse seems appropriate. However, the social acceptability and transparency of scientific research have recently become fundamental issues.

The proactive principle, which emerged from the critical discussion of the precautionary principle, has also found its place in synthetic biology. The proactive principle encourages all parties to actively consider all consequences of an activity, both good and bad while taking precautionary measures against real threats. At the heart of this principle is a commitment to the application of scientific research and technological innovation for the benefit of humanity (More, 2013).

In synthetic biology, a proactive approach would maximize research freedom and commercial benefit to drive innovation and minimize recklessness (Newson, 2011). Proponents of this approach advocate a policy of non-interference in technology development unless there is good reason to suspect that serious physical harm will occur. Proponents advocate minimizing regulation, preferring self-governance instead. However, they support educating the public about the risks and benefits of synthetic biology and advocate for more informed consumers (Race et al., 2012).

Synthetic biology is a cutting-edge technology that differs from other technologies in certain respects and has unique characteristics. This meaning inevitably involves concerns about unknowability and anticipation (Schmidt, 2015). The notion of social and ethical responsibility comes to the fore in synthetic biology, which is both a new field with the potential for continuous transformation and a field of fundamental science and practice (Giese et al., 2015). One of the main concerns of the field is the gap between basic science and practice, which still needs to be adequately addressed. In this context, the problem of uncertainty and foresight in terms of application and outcomes arises. This includes responsibility, responsible development, research, and risk (Giese et al., 2015).

The works of authors such as Hans Jonas have been influential and crucial in terms of discussions and concepts related to the field (Jonas, 1984). For example, Jonas recognized the convergence of biology and engineering before synthetic biology was fully established and made important predictions about the boundaries and future of the field. The ethics of responsibility is a fundamental concept for Jonas and has been crucial in shaping several discussions and concepts in the field (Schmidt, 2015).

The inherently positive meanings attributed to science and scientists with the Enlightenment, such as beneficence, impartiality, and objectivity, and the emergence of positive and negative outcomes in the relationship between technoscience and power in the 20th century, have put on the agenda the need to rethink and expand the concept of responsibility attributed to science and scientists. Since the mid-twentieth century, as the power of technoscience to do both good and harm has become more apparent, it has become clear that the discussion of responsibility in science needs to be broadened. This broadening has meant not only a discussion of external influences and risks but also an assessment of science’s purposes and underlying drivers. This is directly relevant to synthetic biology when considering uncertainties and ethics (Macnaghten et al., 2016).

Developments in technology and synthetic biology should be approached responsibly, based on an empirical, ethical, and epistemological model. The basis of the responsibility discourse is related to the fact that synthetic biology differs from classical basic sciences in several ways. While the basic sciences prior to synthetic biology were fundamentally oriented towards understanding the natural world, the features of synthetic biology, such as redesigning or creating, position it in a different place by taking it beyond basic science. While this feature of synthetic biology constitutes the scientific aspect of the field, the practical aspect of the field has led to a frequent emphasis on concepts such as responsible development, research, and innovation, which include ethical and cultural elements as well as the scientific aspect (Grunwald, 2015).

The responsible research and innovation model represents a novel governance approach that emerged in the 2010s and incorporates many elements of the ELSI (USA)/ELSA (Europe) model (Ethical, Legal, and Social Implications/Aspects) (Zwart et al., 2014; Gregorowius and Deplazes-Zemp, 2016). During its most significant influence, between 2002 and 2012, the ELSI/ELSA model was engaged in several significant scientific initiatives, including the Human Genome Project. It was pivotal in the interaction between technological advances and social processes. In contrast to conventional technology assessment models, these programs addressed ethical and legal issues and conducted interdisciplinary studies in collaboration with different stakeholders (Gregorowius and Deplazes-Zemp, 2016).

Responsible innovation and research aims to assess outcomes and impacts not only after the technology has been developed but also during the technology development process (Gregorowius and Deplazes-Zemp, 2016). It is not limited to ethical reasoning and evaluation. In particular, the epistemological position of emerging technologies and the implications of the knowledge produced in this field in terms of predicting uncertainty are issues that should be considered by researchers working in this field. This means that epistemological issues should also be considered in the context of stewardship. In addition, responsible research, development, and innovation should also address empirically assessable issues related to power distribution, stakeholder and user participation, and governance and communication processes in the field. Therefore, it is suggested that the concept of responsible research and innovation should be addressed in a way that includes ethics as well as epistemological and empirical issues (Grunwald, 2015).

It is emphasized that processes related to responsibility should be handled with democratic participation. Good scientific practice, responsibility to future generations, continuity in thinking about responsibility, and the concept of responsibility should be closely linked to democracy. It is stated that issues related to responsibility should be addressed not only by ethicists but also by interdisciplinary teams, including philosophers, political scientists, social scientists, researchers working on management issues, and especially biologists. In addition to these stakeholders, it is important to work with independent actors outside the scientific community. Only in this way can the question of responsibility be addressed ethically, empirically, and epistemologically in a multidimensional and democratic way (Grunwald, 2015).

Responsible research and innovation is necessary to increase the possibilities of anticipating future risks and taking an ethical position. Recently, empirical studies in the form of designing future scenarios have begun to support this conceptual framework. In this way, it is believed that normative ethical discourses can be generated (Betten et al., 2018). It is also stated that thinking about the future contributes to the possibility of solving problems and transforming the process (Selin, 2008). As a result, expectations and predictions are central to discussions about synthetic biology. In this context, it is possible to see interdisciplinary efforts in the field. However, this must be done through structured institutions with independent and decision-making powers that do not inhibit science and prioritize ethical sensitivities.

Potential applications of synthetic biology in health raise concerns beyond the physical and non-physical, such as the commercialization of synthetic biology, synthetic biology tourism, and access to treatment. There are concerns that synthetic biology will lead to transnational inconsistencies in the regulation of research and healthcare and differences in treatment. As a result of inaccessibility to treatment due to resource constraints or regulatory barriers, it is thought that people will travel to other places to obtain these treatments, encouraging both health and research tourism. If regulations in one jurisdiction are too restrictive or perceived as such, scientists and biotechnology companies may relocate their research activities to a more liberalized area. This would raise concerns about scientific backwardness and economic devaluation of the local biotechnology sector, with economic and political consequences regarding possible ethical objections and appropriate regulation. In addition to intellectual property, the prospect of profits from research on synthetic biology therapies will create pressure to ensure a receptive market for these therapies. This pressure will encourage active marketing of health applications of synthetic biology, which will also influence public expectations, understanding, and attitudes towards these technologies and synthetic biology in general (Chan, 2018).

A vibrant content under this theme will open the door to meaningful discussions. This is undoubtedly a richness in terms of bioethics. In this context, Newson’s mention of the need for professional ethics in the field is important (Newson, 2011). By its very nature, synthetic biology consists of researchers from different disciplines. In this context, researchers in each discipline will likely have different codes of conduct or expectations of what constitutes responsible research. While researchers need to evaluate the results of their disciplines, a more holistic perspective and evaluation will become more critical as synthetic biology develops (Newson, 2011).

## 5 Conclusion

Ethical debates on synthetic biology have been conducted on various issues. In this study, ethical debates are visible as the moral positions of synthetic biology products, the meaning of life, metaphors, the use of knowledge and expectations, concerns, and ways of solving problems. According to the findings, there is a naming problem for the synthetic biology product. The distinction between a synthetic biology product as a machine or a living being makes a significant difference. Such labeling will affect the moral value attributed to the synthetic biology product. There are two different views of this connection. One is that living organisms have intrinsic value. This value is independent of whether or not the living organism is produced for the benefit of human beings; it is related to vitality. The other view is that these living things do not have intrinsic value but only instrumental value. This value is instrumental to human beings, and using these organisms for human benefit can be justified.

The question of what synthetic biology’s product is as an entity has been a crucial issue in the ethical debate. In this context, the entities’ artificiality, syntheticity, or living organisms are the main determinants of the debate. This inevitably affects the moral position of these entities. Synthetic biology is not only a practical field but also a field in which scientific knowledge is produced. The production process and the meaning of this knowledge are also critical.

The knowledge produced provides benefits; on the other hand, it can be misused, raising the issue of dual-use. Again, issues such as this knowledge’s fair allocation and commercialization are ethically significant.

What distinguishes synthetic biology from other technologies is its uncertainty. In addition to the expected benefits, synthetic biology can potentially create risks, especially to human health and the environment. In this context, risks and possible negative consequences are highlighted in synthetic biology and ethics discussions.

Given the interdisciplinary nature of synthetic biology, while the different approaches and goals of researchers from different disciplines are important for the field’s development, a professional ethics for synthetic biology is needed. The field’s development will bring the concept of professional integrity to the forefront. Interdisciplinary work will also help to reduce the gap between the development of new technologies and the development of rigorous and responsible policies.

Public support should also be obtained to successfully and rapidly realize the potential of synthetic biology. To this end, it is important to educate the public. Transparency towards society in addressing the potential risks of synthetic biology is necessary for society to accept and support this technology. Scientists have a responsibility to society in the face of potential risks. Since the researchers first obtain the research results, those working in the field must inform society about practical or abusive uses.

Given the speed of development and the dynamic nature of the field, it is inevitable that there will always be various ethical concerns as well as potential benefits. Moreover, reaching a final decision on these concerns is impossible. A multidimensional method should be applied to the problems that arise or may arise. In this context, one of the most critical factors is society; while society should be adequately informed about the processes, its contribution should also be sought.

As this research underscores, navigating ethical debates in synthetic biology requires a multidimensional approach that fully embraces the field’s complexity. For example, the Synthetic Yeast Project not only showcases global scientific collaboration but also highlights the importance of addressing ethical concerns in parallel with scientific development, resulting in guidelines that help mitigate biosafety risks (Synthetic Yeast, 2023). Similarly, the Human Practices component of the iGEM (International Genetically Engineered Machine) competition requires projects to consider societal impact from the outset, encouraging socially responsible and widely accepted innovations (iGEM, 2023).

In addition, the Gene Drive Research Collaboration is an example of how multi-stakeholder engagement can lead to more comprehensive risk assessments and better-informed environmental and public health decisions (The Foundation for the National Institutes of Health, 2023). The EU’s Horizon 2020 program, with its emphasis on involving societal actors, illustrates the benefits of research agendas that reflect public values and expectations (European Union, 2024). This inclusive approach fosters trust and transparency, making scientific progress a shared endeavor.

Seven these initiatives demonstrate the significant benefits of collaborative, interdisciplinary efforts that advance the field and ensure that ethical considerations are integrated from the outset. However, these collaborations face challenges, such as reconciling diverse viewpoints and managing complex logistics. Recognizing these hurdles is critical, as it underscores the ongoing need for adaptive strategies and open dialogue to address the ethical dimensions of synthetic biology effectively.

## Data Availability

The original contributions presented in the study are included in the article/Supplementary Material, further inquiries can be directed to the corresponding author.
