# Supplementary material for: The view of synthetic biology in the field of ethics: a thematic systematic review
Source: Front Bioeng Biotechnol. 2024 May 28;12:1397796. doi: 10.3389/fbioe.2024.1397796 (PMC11165145; doi:10.3389/fbioe.2024.1397796)
Supplement: Supplementary file 1 [file Table1.DOCX]

Supplementary Material

The View of Synthetic Biology in the Field of Ethics: A Thematic Systematic Review

Ayşe Kurtoğlu^1*^, Abdullah Yıldız^1^, Berna Arda^1^

*** Correspondence:** Ayşe Kurtoğlu: [akurtoglu@ankara.edu.tr](mailto:akurtoglu@ankara.edu.tr), [aysekurtoglu87@gmail.com](mailto:aysekurtoglu87@gmail.com)

**Supplement 1. Detailed analysis of the publications included in the study**

This table presents the results of a thematic analysis focused on ethical considerations in synthetic biology. It categorizes key debates, conceptual frameworks, and proposed solutions from a curated collection of scholarly articles. The analysis aims to systematically capture the dynamic ethical landscape and facilitate a nuanced understanding of the challenges and opportunities presented by advances in synthetic biology.

| **Author, Year** | **Ethical Debate** | **Key Concepts** | **Conclusions and/or Recommendations** |
| --- | --- | --- | --- |
| Bhutkar, 2005 | In the reductionist approach, genes are the basis of living organisms, and constructing a synthetic life form with a set of genes can be considered a living organism. However, more theological views argue that life is not just a set of genes but has non-physical aspects. This is especially true for higher organisms.  Ethical debates in biotechnological research become visible when mammals or higher organisms are affected. With the development of synthetic biology, synthetic or minimal genomes can be envisioned for mammalian species such as mice. Is it ethically appropriate for scientists to conduct this level of research? What is the benefit of this research to society?  What are the ethical and moral responsibilities of researchers in this field? Should a researcher have a moral responsibility to restrict work on organisms to ensure they do not compromise public safety? Can this be done in a new field where there are many unknowns? How should a researcher control information about findings that could be used to produce synthetic organisms that are harmful to humanity or the environment? | Instrumental value  Intrinsic value  Professional ethics | A set of ethical principles can be proposed for synthetic biology research:  (1) Clear articulation of instrumental and intrinsic value: Synthetic biology productions will have instrumental value for humanity. Scientists and ethicists should also consider any intrinsic value that these products may have as the classification of the resulting products moves to higher levels (synthetic elements, networks, organisms, systems).  (2) Step-by-step expansion of the universe: As part of advancing technology, one must be aware that as one moves to higher levels, there are additional unknowns. As researchers move to the next level of classification (synthetic elements, networks, organisms, systems), the parts at each classification level should be well characterized, and caution should be exercised until their effects are determined. |

| **Author, Year** | **Ethical Debate** | **Key Concepts** | **Conclusions and/or Recommendations** |
| --- | --- | --- | --- |
| Preston, 2008 | In synthetic biology, genome modification is conducted in a highly controlled and specific manner, primarily through DNA sequences with known properties known as biobricks. Many synthetic biology applications involve inserting these synthetically engineered DNA sequences into existing single-celled organisms. The aim is not necessarily to modify the established properties of existing bacteria but rather to create a new organism that possesses the desired properties.  Synthetic biology products do not borrow genetic functions from genomes produced through the evolutionary process. Instead, these products aim to modify the evolutionary process and existing genomes for specific purposes.  A concerning aspect is that this artificial approach differs significantly from the basic principles of Darwinian evolution, particularly the transmission of traits from one generation to the next through modification.  When synthetic biologists construct a new genome, de novo using biobricks sequences, they break the causal continuity with the evolutionary past. In synthetic biology, all lineage traces from naturally selected ancestors are removed. Although synthetic biology products contain nucleic acids, the artefacts created do not derive their genetic sequences from the evolutionary process. | Artificiality  Changes in the  evolutionary process | ---- |

| **Author, Year** | **Ethical Debate** | **Key Concepts** | **Conclusions and/or Recommendations** |
| --- | --- | --- | --- |
| Bedau et al., 2009 | Protocell research does not require laboratory safety or risk management. Protocell development should have ethical, social, and regulatory control points (CPs):  Ensure that ethical, social and ultimate regulatory implications are considered in systematic and advanced research on protocell synthesis.  The technical feasibility of protocells is an essential social and ethical checkpoint, indicating that all major scientific barriers to making autonomous cells have been overcome.  The creation of the first autonomous protocells involves developing a self-assembling and self-reproducing chemical system with containment, metabolism, and programmability properties.  Protocells that can survive outside the laboratory can potentially harm humans and the environment. The ability to survive in the outdoor environment should prompt a reassessment of the regulatory and containment standards for protocells.  Releasing protocells outside of the laboratory is unique because these cells will be in direct contact with other life forms, including humans, which has a social significance.  Toxic or infectious protocells will stimulate the need for appropriate safety regulations. Using protocells outside the laboratory for medical or environmental applications will further increase such a risk. | Protocell | Scientific results should be published openly and rapidly. Social, cultural, and religious concerns about protocells should be discussed with the public. In order to assess the social, ethical and legal aspects of protocells, part of the research funding should be dedicated to these issues.  Training on the benefits, risks, uncertainties, and best practices of protocell research should be a regular part of university curricula.  Once technically accessible, intellectual property rules should be re-evaluated for compatibility with the characteristics of these cells.  The potential for malicious use of protocells should be assessed.  Appropriate oversight bodies for fully autonomous applications should be established prior to the existence of protocells, and these bodies should be re-evaluated at each subsequent control point.  Regulatory authorities should develop safety-level classifications and protocols for safely using autonomous protocells in the laboratory.  A comprehensive plan for safety mechanisms should be developed before protocells are developed; a range of safety mechanisms should be defined. Thoroughly tested safety mechanisms and quality assurance measures should be in place before protocells are released outside the laboratory and should be re-evaluated at subsequent control points so that these mechanisms can be adjusted and improved. |

| **Author, Year** | **Ethical Debate** | **Key Concepts** | **Conclusions and/or Recommendations** |
| --- | --- | --- | --- |
| Dabrock, 2009 | Synthetic biology is not about playing God. The power to create is divine and cannot be claimed by humans.  Similarly, the suspicion that this branch of science points to the failure of man to recognize his position and duties within creation is not true.  The idea is that synthetic biology crosses a fundamental boundary in human cultural memory, intruding into a domain thought to belong exclusively to the divine. | Playing God | Systematic theology and theological ethics can help to demythologize the assessment of synthetic biology. Theological creation and the concepts of sin, this field that it has not conquered a so-called sacred, divine space.  Synthetic biology is no more deontologically blameworthy than any other conceivable technology, human action or institution.  For all known and future biotechnologies, risks regarding resilience, clarity of purpose, social relevance and safety can be assessed. |

| **Author, Year** | **Ethical Debate** | **Key Concepts** | **Conclusions and/or Recommendations** |
| --- | --- | --- | --- |
| Deplazes et al., 2009 | Synthetic biology represents a new phase in human actions. The ethical and philosophical implications of defining created beings as organic beings or machines are inevitable.  Synthetic biologists face the problem of naming what they make. The nomenclature of machine and organism implies important differences. This difference inevitably leads to a distinction about the moral status of beings.  There are many theories about the moral status of living organisms. Within these discussions, living organisms are often considered subjects of moral consideration. The main debate focuses on the type of value that should be attached to living organisms, whether intrinsic, instrumental, or a combination of different ethical perspectives.  For some ethicists, living organisms have intrinsic value regardless of whether they are useful for human purposes. For other philosophers, lower categories of living beings have no intrinsic value, but they have instrumental value to humans and can be used for human purposes.  If a living being is considered to have intrinsic value, then its use for human purposes must be justified. Conversely, if a being is considered to have only instrumental value, its utility in serving human needs is paramount. This distinction is crucial in determining how different life forms are ethically viewed and used. | Living organism  Machine  Intrinsic value  Instrumental value | The terminology used to describe synthetic biology products as either machines or organisms carries weight beyond mere metaphor.  While there are certain similarities between machines and organisms, their fundamental differences have significant philosophical and ethical implications. These distinctions play a crucial role in determining whether these products are viewed through the lens of intrinsic or instrumental ethical values. |

| **Author, Year** | **Ethical Debate** | **Key Concepts** | **Conclusions and/or Recommendations** |
| --- | --- | --- | --- |
| Douglas et al., 2010 | Synthetic biology is a rapidly developing field with the potential to benefit humanity significantly. For this reason, it is at the center of interest in many disciplines.  The expectations of the bioethics field and its contribution scope still need to be clarified. It is important to identify and understand this.  Prominent ethical debates on synthetic biology have focused on playing God, the blurring of the distinction between life and machines and the moral subject in this regard, the misuse of knowledge (dual use and bioterrorism), and intellectual property.  In this process, bioethics should consider not only the production processes of knowledge but also the meaning and distribution of the knowledge sought. In this context, the scope of bioethics should also be broadened. This discussion is conceptualized in terms of the ethics of knowledge. | Dissemination of knowledge  The meaning and ethical implications of the knowledge produced  Ethics of knowledge | Bioethics should extend its focus beyond the processes of knowledge production to include the dissemination and meaning of knowledge. It is essential that knowledge generation realistically addresses risks and incorporates effective internal control mechanisms for information management. Engaging with knowledge producers about the potential risks associated with their work is essential.  Government oversight should also be a key component of this framework to maintain ethical standards. All of these processes must be carefully balanced to avoid hindering the progress of knowledge production. In navigating these complex issues, the role of ethicists is crucial in clarifying the arguments and value perspectives involved, thereby contributing to a more ethically informed approach to synthetic biology. |

| **Author, Year** | **Ethical Debate** | **Key Concepts** | **Conclusions and/or Recommendations** |
| --- | --- | --- | --- |
| Saukshmya  et al., 2010 | The rapid development of synthetic biology has outpaced the development of responses to its ethical and social implications. These issues are multifaceted, encompassing both the potential benefits of synthetic biology and its potential adverse effects on the environment and wildlife.  Concerns about biosecurity and the risk of bioterrorism are paramount, as are the implications of commercializing synthetic biology products. The complexities of intellectual property rights, particularly in balancing commercial interests with the public good, also require careful consideration. | Risk of harming wildlife and the environment  Biosafety  Biosecurity  Bioterrorism  Dual use dilemma  Intellectual property  Commercializa- tion | A responsible scientific approach that understands societal concerns needs to be developed.  Researchers should be aware of the societal implications of their research.  Stakeholders should be involved and contribute to policy development.  Biosecurity regulations should be put in place.  Risk assessment systems should be activated.  Intellectual property rights should be regulated.  Governments, researchers, scientific institutions and users should support risk-reduction processes. |

| **Author, Year** | **Ethical Debate** | **Key Concepts** | **Conclusions and/or Recommendations** |
| --- | --- | --- | --- |
| Mori et al., 2011 | The unintended and unexpected consequences of synthetic biology are a major concern. Introducing artificial biological systems into the environment risks ecosystems and human health (biosafety), mainly if these organisms adversely affect natural systems or transfer genes to native species. In addition, the unpredictable evolution of these systems could inadvertently lead to the development of human pathogens or harmful substances.  There are also concerns about the deliberate misuse of synthetic biology, such as replicating existing pathogens or engineering new, more virulent strains. Such scenarios highlight the need for stringent biosecurity measures.  Amidst these concerns, ethical debates have emerged, particularly in Western societies, about the permissibility of playing God with synthetic biology. These debates often focus on the implications of creating artificial life forms, with some fearing the potential creation of monster-like beings, raising profound questions about the limits and responsibilities of scientific innovation. | Biosafety  Biosecurity  Playing God  Proactive approach  Precautionary approach | Regulatory arrangements in the early stages of trials and the regulation of laboratory products should be coordinated internationally.  Voluntary action should be taken by individual organizations before countries create different regulatory regimes.  Several options could be proposed, such as customer screening by DNA synthesis companies, training of scientists in biosecurity issues, establishment of a professional society for synthetic biology, and development of a laboratory biosecurity manual.  Academics, artists, companies, government organizations and citizens working in the social sciences should be seen not only as contributors who support the progress of research by learning about results or mediating communication with society, but also as potential collaborators who can influence scientific knowledge. |

| **Author, Year** | **Ethical Debate** | **Key Concepts** | **Conclusions and/or Recommendations** |
| --- | --- | --- | --- |
| Newson, 2011 | Physical harm, which encompasses biosafety and biosecurity issues, is a major challenge. Biosafety refers to the adverse effects of synthetic organisms on the environment or humans, but what is safe and who determines this is controversial. Biosecurity concerns include the potential misuse of synthetic biology for bioterrorism and the dual use dilemma. Non-physical harms involve ethical considerations about the purposes and applications of synthetic biology how they might affect the well-being of individuals or communities. Central to this is the role of humans in the creation of new organisms and their interaction with the environment. As applications become complex, assessing the moral status of new beings and reassessing our understanding of life, nature, and creation is imperative. Questions about the acceptability of creating life are central to these discussions.  Professional ethics in synthetic biology also warrant attention due to the field’s interdisciplinary nature. Professionals from different disciplines bring different expectations and codes of conduct, leading to potential conflicts and challenges. As the field develops, maintaining professional integrity will become increasingly important.  The commercialization of synthetic biology raises questions about patenting and intellectual property rights. The debate about who owns the rights to synthetic biology products and the impacts of these rights on research and innovation is an ongoing concern. | Biosecurity  Biosafety  Dual use  Professional ethics  Moral status  Fair distribution  Commercial benefit | Harm from synthetic biology can be mitigated through appropriate regulation.  There are different models, such as legislation, codes of conduct, professionalization or self-regulation. Each of these models has different positive and negative aspects. A combination of these models is necessary for synthetic biology.  There should be negotiations on how the processes will evolve to share the benefits fairly.  Research on the ethical, legal and social aspects of synthetic biology (1) does not need a new branch of bioethics; (2) should focus on new concepts and problems; (3) should be interdisciplinary. |
| **Author, Year** | **Ethical Debate** | **Key Concepts** | **Conclusions and/or Recommendations** |
| Charpa, 2012 | From a meta-ethical point of view, when synthetic biology is compared to the golem metaphor, this metaphor affects our moral judgements and assumptions.  In virtue ethics, creating a golem is not easily acceptable because the creator may lack the skills to control it easily, such as prudence and responsibility.  For consequentialists, synthetic biology will cause unpredictable disasters in the context of the golem metaphor.  From a categorical (Kantian) point of view, this metaphor does not see synthetic biology as generally permissible.  However, relying heavily on metaphorical discourse in ethical analysis can be problematic. While metaphors such as that of the golem can illustrate complex ideas, they also risk oversimplifying and potentially misrepresenting the nuanced ethical landscapes of fields such as synthetic biology. In reality, moral ambiguity can be unfairly transformed into moral unacceptability through such reductive narratives. | The significance of metaphors in terms of ethics is that, despite utility and good purpose, unpredictability and future risks  Prudence  In terms of metaethics, the possibility that metaphors overemphasize risks and make ethical discussions unproductive | The golem metaphor hinders ethical debates on synthetic biology in several ways.  While consequentialism, the dominant ethical approach in synthetic biology, is relatively proaction, the golem metaphor tends to be restrictive, focusing on protection and risk prevention rather than promoting appropriate and effective consequentialist approaches.  Good ethical analysis cannot be done if moral analysis focuses on discourses like the golem metaphor. This is because metaphors increase the likelihood that morally ambiguous issues will become morally unacceptable. |

| **Author, Year** | **Ethical Debate** | **Key Concepts** | **Conclusions and/or Recommendations** |
| --- | --- | --- | --- |
| Glick, 2012 | In Judaism, humans are permitted to use their intelligence, creativity and physical abilities to develop and improve the world in which they live.  Synthetic biology scientists work to make the world a better place. The term playing God is an unfair and unfortunate pejorative. The fear of playing God is not valued in the Jewish tradition.  Biosafety concerns are real, and all the characteristics of created organisms are not always predictable. Even when the current situation is well understood, change is always possible.  In the Jewish tradition, humans are commanded to be creative and take initiative. However, man is warned against hubris and the assumption of omniscience and absolute power. | Biosafety  Playing God | Scientists need the support of society to successfully and rapidly realize the potential of synthetic biology. It is essential to educate the public. A proactive effort is needed to explain what scientists are doing and to make it clear to the public that their efforts have the potential to help solve many of the world’s problems.  Great attention to biosafety is crucial to maintaining a positive public image of synthetic biology. Any mishap, even a relatively minor one, can have a devastating impact on the field. This is one reason for scientists to be vigilant.  When playing with the DNA alphabet, it is important to ensure that trivial changes do not lead to unintended consequences. |

| **Author, Year** | **Ethical Debate** | **Key Concepts** | **Conclusions and/or Recommendations** |
| --- | --- | --- | --- |
| Heyd, 2012 | The ethical issue of synthetic biology is based on the distinction between the natural and the artificial. The distinction between the natural and the artificial is the basis of philosophical opinion. When the artificial significantly dominates the natural, a concern arises; the rhetorical expression of this concern is playing God.  It is a divine prerogative to attempt to create something out of nothing, and man’s attempt to emulate God is unacceptable; it is perceived as arrogance. Although man is the only craftsman in the world, he must realize that the limits of his natural place bind him. Possessing the power to create, man is and must remain a product of the evolutionary process, not its director.  Despite the dramatic nature of synthetic biology, there is nothing ethically new about it. All technological developments since the invention of the wheel are potentially double-pronged. However, this does not mean we should be indifferent to laboratory work involving extreme risks.  Beyond the explicit and widely accepted principle of freedom of scientific inquiry, pursuing knowledge must be a social activity, ultimately dependent on or constrained by social norms and values. | Natural/artificial distinction  Dual use  Playing God | The main methodological and social problem in the ethical regulation of synthetic biology research is that scientists need to learn more about research risks. The benefits and harms of research can be assessed through scientific developments.  Researchers have an essential responsibility to society for potential risks. As they are the first to receive the research results, it is the responsibility of those working in the field to inform the public about synthetic biology’s practical uses and misuse.  Scientists should also exercise self-control over their work before it is regulated. |

| **Author, Year** | **Ethical Debate** | **Key Concepts** | **Conclusions and/or Recommendations** |
| --- | --- | --- | --- |
| Holm, 2012 | The cell or other living things establish a normative organization for their survival, which can be justified by teleological approaches.  The living being is in a normative organization for survival in interacting with the environment. In this case, the realization or following of certain things ensures the continuation of life. Thus, the system it has for its continued existence can be interpreted as norms for its activities. If it does not fulfil these conditions, its existence becomes impossible.  In terms of the organizational approach, continued existence is sufficient for teleology and normativity. In this context, the normative and teleological structure as an organization has a moral significance beyond the biological interest in morality. | Definition of artefactual organisms  Moral status of artefactual organisms | The normative and teleological approach, necessary for the unique survival of biological life based on organization, is a stronger argument for morality than the biological interest approach. |

| **Author, Year** | **Ethical Debate** | **Key Concepts** | **Conclusions and/or Recommendations** |
| --- | --- | --- | --- |
| Race et al., 2012 | Non-physical harms can be further divided into two sub-categories: issues that primarily concern those directly involved in synthetic biology, such as fair distribution of benefits, control of property, and the tools needed for synthetic biology, and broader ethical issues that, while still relevant, may not be the primary focus of practitioners, including concerns about overarching moral status.  Physical harms consist of issues related to the risks and consequences of synthetic biology, including health, safety and environmental factors; non-physical harms consist of moral values, moral consequences, public welfare and social justice.  Proponents of synthetic biology applications take a proactive approach. According to this approach, new technologies should not be interfered with unless there is good reason to suspect they will cause serious physical harm. Conversely, the precautionary approach generally opposes progress with significant potential for harm. It advocates strong regulation, regular monitoring, transparent public consultation on risks and benefits, and society’s active participation in shaping the explored field’s development.  Many of the concerns related to space exploration overlap with those identified for terrestrial applications of synthetic biology on Earth. Additional space issues include synthetic biology, space law, planetary protection, cross-contamination, space microbiology, risk assessment, decision-making infrastructure, long-term human missions, terraforming, and the potential discovery of extraterrestrial life. | Physical harm  Non-physical harm  Proactive approach  Precautionary approach | A review of past experiences and emerging technologies may help consider the short and long-term impacts and moral implications of possible synthetic biology applications for use in space.  Scientists should be aware that political and societal issues will be addressed incrementally as they seek to integrate scientific advances into decision-making processes.  As researchers continue to explore the various benefits of synthetic biology in the coming years - on Earth and in space - they must also be aware that legitimate questions remain in important areas beyond the scientific domain.  Work with other disciplines to reduce the gap between developing new technologies and creating thoughtful and responsible policies. |
| **Author, Year** | **Ethical Debate** | **Key Concepts** | **Conclusions and/or Recommendations** |
| Rager-Zisman, 2012 | Synthetic biology offers advanced technologies for the deliberate production of organisms capable of producing biological weapons. This means that published scientific information can be misused and raises dual use concerns.  Reconstituted microorganisms may have increased virulence and infectivity, be difficult to detect, or pose unintended risks to humans and the environment.  The most commonly cited ethical issues related to synthetic biology are biosafety and biosecurity. | Dual use  Biosafety  Biosecurity | Ethical and legal analysis should be sought early in synthetic biology, and these discussions should involve experts in ethics, law and technology.  Scientists take responsibility for biosecurity as part of their research, especially when conducting research with dual use concerns. |

| **Author, Year** | **Ethical Debate** | **Key Concepts** | **Conclusions and/or Recommendations** |
| --- | --- | --- | --- |
| Thompson, 2012 | Rapid developments in synthetic biology enable its use in various commercial fields, as well as in medicine, pharmaceuticals and biofuels. The regulations in these different areas are distinct. In this context, the application principles of medical ethics alone may not be sufficient.  Focusing on medical ethics alone can lead to overlooking practices in other areas and cause problems, especially in developing countries.  While medical ethics issues such as stem cells have been at the forefront of discussions, issues related to environmental ethics, agriculture and the philosophy of technology should be addressed.  In developing synthetic biology products useful in areas such as agriculture or medicine, attention should be paid to the ecological problems they may cause. The development of biofuels can be justified in reducing the use of fossil fuels. However, how this will affect the climate and environment differently should also be assessed. Therefore, discussions on synthetic biology need a broader perspective than the relatively narrow medical ethics perspective on technological developments. | Synthetic bioethics | Academic ethics or medical ethics only focus on a limited human dimension of the issues related to synthetic biology. However, with its commercial and economic implications, synthetic biology may have wider implications. In this context, there is a need for broad ethical perspectives and discussions that go beyond traditional medical ethics. |

| **Author, Year** | **Ethical Debate** | **Key Concepts** | **Conclusions and/or Recommendations** |
| --- | --- | --- | --- |
| Bensaude Vincent, 2013 | Ethical debates about synthetic biology are concerned with the risks that may arise in the future. It includes well-established issues such as biosafety, biosecurity, biohacking, intellectual property and social justice.  Synthetic biologists should develop prospective technical solutions to avoid risks. In this context, in addition to traditional containment measures in the laboratory to prevent the spread of synthetic organisms and the risk of contamination of wildlife, it is necessary to produce synthetic organisms that depend on a specific substance not found in nature or that synthesize unnatural nucleic acids such as XNA.  Access to and dissemination of synthetic biology resources is about enabling innovative developments in synthetic biology through open access and preventing the increase of global inequality through international dialogue.  Given the scientific uncertainty, the diversity of actors involved in synthetic biology, and the scientific, industrial and geopolitical interests, the most effective governance should encourage continuous reflexivity among stakeholders rather than a rigid regulatory regime. | Biosecurity  Biosafety  Social justice  Intellectual property | At the boundary between the natural and the artificial, the animate and the inanimate, how objects are designed in laboratories inevitably challenges the fundamental divisions of modern Western culture. This raises profound questions about humanity’s place and role in nature and our relationship with animals and the environment.  Under the umbrella of synthetic biology, we should distinguish between different organisms based on how they exist rather than on potential risks alone. This approach takes into account the intrinsic qualities of synthetic organisms.  The concept of ‘responsibility’, as advocated by the synthetic biology community, does not adequately address the ethical concerns raised by this new technology. Without doubting the good intentions of synthetic biologists, their approach to ethical issues seems strongly opposed to public involvement or intervention in their field. In addition, their focus on risk issues promotes the idea that societal and environmental challenges can be solved by technological means.  Dealing with uncertainties in synthetic biology also requires strengthening the link between the scientific community and civil society, fostering dialogue and collaboration to navigate these complex ethical landscapes. |

| **Author, Year** | **Ethical Debate** | **Key Concepts** | **Conclusions and/or Recommendations** |
| --- | --- | --- | --- |
| Boldt, 2013 | Despite the potential risks and harmful impacts, synthetic biology can be considered a beneficial addition to biodiversity. Such an increase in biodiversity is ethically justifiable or desirable. In this context, an understanding or investigation of the intrinsic value of biodiversity would be helpful to analyze such views. The intrinsic value of biodiversity should be analyzed when considering whether there is a moral obligation to produce synthetic organisms to increase biodiversity.  The two approaches to biodiversity attempt to show whether there is a moral obligation to synthesize organisms to increase biodiversity. The main difference between these approaches lies in their perspectives on human knowledge acquisition and its ethical implications. The first approach is the admiration stance, which rejects the creation of synthetic biology products and emphasizes the value of natural biodiversity. The second approach is the kinship stance. It assigns value to synthetic and natural species or organisms.  Creating a new species is sometimes an ethical necessity, particularly when it contributes positively to biodiversity. However, this perspective tends to favor modifying existing organisms and establishing a relationship with them rather than synthesizing entirely new organisms from scratch. Based on these approaches, there cannot be an inherent moral responsibility to increase biodiversity. Therefore, synthesizing organisms solely to increase biodiversity cannot be considered a moral duty according to these approaches. | Moral obligation  Intrinsic value of biodiversity | The moral obligation to synthesize organisms to increase biodiversity cannot be justified by appealing to the admiration or kinship stance, two approaches that attribute intrinsic value to biodiversity. |
| **Author, Year** | **Ethical Debate** | **Key Concepts** | **Conclusions and/or Recommendations** |
| Boldt, 2013 | According to Arendt’s concept of fabrication, a created product can exist for a long time. The process is continuous and ongoing; the fabricated product is new and durable. However, it is also susceptible to damage and can be destroyed by its creator.  In the fabrication concept, the human being can be positioned as a designer or a fabricator. In this position, humans are constructed as having the technical knowledge of production and the ability to control and predict the product.  Synthetic biology generally emphasizes fabrication and the discourse around it. The product is conceived as a machine whose properties are known and predicted. The term creation is also used for the fabrication process. In this context, humans are seen as homo creator. The discourse of creating life is often used concerning synthetic biology.  From an action perspective, there are three main problems with the fabrication approach commonly used in synthetic biology: synthetic biology may overestimate its ability to create new organisms and to do so reliably; synthetic biology’s means of assessing and limiting the safety of the new products it creates may be limited; synthetic biology itself may not be able to establish criteria for the intrinsic value of things about the meaning of life.  The product and production of synthetic biology is an unpredictable, unlimited act from the perspective of action. | Viewing synthetic biology as an activity of creating technological innovation  Meaning of a synthetic biology product  Fabrication | ---- |
| **Author, Year** | **Ethical Debate** | **Key Concepts** | **Conclusions and/or Recommendations** |
| Braun et al., 2013 | With synthetic biology, we have moved from a position limited to quantitative changes in nature to a dimension that can make qualitative changes. This is a paradigmatic shift in the anthropological interpretation of human beings.  Cultural and religious metaphors play an important role in understanding human technological change among humans. In this respect, the substitution of one metaphor for another is significant. These are not simple discourses; they are important in understanding the view of reality in terms of experience and action.  The discourses of playing God or creating life attributed to synthetic biology in the context of Christian theology are not precisely equivalent to the concept of God’s creation. Creation or generation in synthetic biology is secondary to what exists. In this respect, the human attribute of creativity is never authentic in the sense of full divine creativity compared to theological creativity.  Awareness of the nuances of theological ethics allows for an appropriate and sensitive handling of the implications of these studies for society and science. In this context, a sensitive approach that includes theological ethics does not entirely ignore the possibilities of human creation but also ensures a responsible approach. Human beings who are aware that they are created can recognize that they are involved in the act of creation. This means that human creations are religiously justifiable. It is crucial to recognize that human action can affect other things. | Theological-ethical approach to synthetic biology  Playing God | Theological ethics should also be considered in discussions of synthetic biology. This field can provide clarification and understanding of the human act of creation and the metaphorical discourses related to it, and it can also show that human creation is religiously justifiable.  Furthermore, human creation as a created being differs from divine creation, and it is an important value for humans to approach technology with a sense of responsibility. |
| **Author, Year** | **Ethical Debate** | **Key Concepts** | **Conclusions and/or Recommendations** |
| Douglas et al., 2013 | There are three value discourses regarding the moral significance of creating artificial life. These are the claim that to create an artificial being is playing God; the claim that to create an artificial being will result in a reduction that undermines the particular moral values of life; and the concern that the functions of artificial organisms will be ambiguous, and hence their moral status will be ambiguous and unclear.  In the first discourse, the idea that the creation of life has a direct, morally negative implication needs to be clarified; in the second, the possibility that the creation of artificial beings leads directly to a reductionist attitude must be clarified. In the third discourse, the indeterminate properties of organisms may be evolutionarily variable; in this respect, their moral status will already be related to their properties.  These statements cannot be justified by the view that the creation of artificial life is not derived from existing life forms of organisms.  What is morally distinctive is not how the organism is created but the properties that the created being possesses. | Meaning the creation of artificial life  Playing God  Moral status | The ability to create life forms that cannot exist spontaneously raises profound moral questions about their interests, moral status and the risks they pose to other beings.  In considering questions of moral significance, the issue is not how life was created but what characteristics these created beings have. |

| **Author, Year** | **Ethical Debate** | **Key Concepts** | **Conclusions and/or Recommendations** |
| --- | --- | --- | --- |
| Heavey, 2013 | Synthetic biology is not socially detrimental to the dignity of life. The fact that it is applied in ways that are dangerous to life or lead to some negative attitudes does not mean that synthetic biology is a challenge to the dignity of life. However, the fact that humanity has advanced to the point of creating artificial life reflects the dignity of humanity and of life itself.  The discourse of playing God is not unique to synthetic biology; it has been raised against scientific advances such as genetic engineering, anaesthesia, organ transplantation, artificial contraception, and the diagnosis of brain death. A significant number of mainstream religious thinkers also believe that synthetic biology in itself does not have the creative role of God. | Dignity of life  Playing God  Deontological approach | It is not enough to apply different ethical tools to an important question if these tools give conflicting answers. In this context, more than one ethical approach is required for a complete assessment of synthetic biology.  Applying a single ethical approach to synthetic biology is like applying a failed algorithm to a complex problem. An appropriate mix can provide a useful, if imperfect, heuristic, and ethically it can get us closer to the truth. |

| **Author, Year** | **Ethical Debate** | **Key Concepts** | **Conclusions and/or Recommendations** |
| --- | --- | --- | --- |
| Holm, 2013 | Paley organism refers to both being biological and being created by humans. These organisms are endowed with a moral status based on their benefits and interests that can be attributed to their location.  The maintenance of biological functionality is a necessity for these organisms. Maintaining the organism’s existence means that the act of the acting organism also has normative significance. The moral status of self-sustaining synthetic organisms needs to be assessed.  One of the fundamental moral distinctions between living organisms and other organisms that are products of production is that organisms act in their interest and for their benefit. A moral status can be ascribed to synthetic organisms in the light of maintaining appropriate functions for their survival. | Teleology  Moral status  Paley organisms | Paley organisms can be assigned appropriate functions based on their self-sustaining organization. |

| **Author, Year** | **Ethical Debate** | **Key Concepts** | **Conclusions and/or Recommendations** |
| --- | --- | --- | --- |
| Kelle, 2013 | A better understanding of the mechanisms of regulatory systems, such as the nervous, immune or endocrine systems, also opens up the potential to misuse biologically active chemical compounds that can target these systems with greater specificity. Synthetic biology will also have the potential for dual use and misuse once issues of standardization of parts and modules are resolved.  Governance measures for dual use extend to the education and training of synthetic biologists who are working and/or will work in the field, codes of conduct, guidelines, regulations, national and international legal texts. | Dual use  Governance measures  Precautionary principle | A matrix of governance measures for potential dual use is proposed, including five different intervention points (the principal investigator, the project, the premises, the provider and purchaser, and the public). This matrix is intended to facilitate identifying and discussing governance proposals or existing and implemented measures for dual use and explore their precautionary principles.  A more systematic precautionary governance approach should be adopted for the dual use of synthetic biology. There is a need for a structured discourse at institutional, national and international levels on the scope, content and processes of this systematic and sustainable governance approach. |

| **Author, Year** | **Ethical Debate** | **Key Concepts** | **Conclusions and/or Recommendations** |
| --- | --- | --- | --- |
| Link, 2013 | Synthetic biology blurs the distinction between animate and inanimate, natural, and artificial. The concern that this distinction will disappear is linked to the creation of higher forms of life.  For synthetic biology to be morally questionable, one must first focus on the intrinsic value of life and how synthetic biology might conflict with these values.  The accusation of violating the dignity of living beings by creating them for a specific purpose confuses meaning. Designing and producing organisms does not mean instrumentally treating them or failing to respect their telos. In the specific case of synthetic biology, the intrinsic value of life is not undermined.  The idea that synthetic biologists will have a powerful tool at their disposal raises concerns that they can play carelessly with nature. As the potential impact of these new technologies grows, the risks appear enormous and incalculable and are seen as tantamount to playing God. The idea of playing God, however, is not a new discourse. | Intrinsic value  Playing God | A community’s path is a matter of collective and political choice rather than a matter of ethical expertise. It is, therefore, the responsibility of policymakers to ensure that society decides to what extent it will approve research and how it will fund it.  A public dialogue on synthetic biology should be encouraged, and many questions should be clarified. At this point, philosophers can also contribute by clarifying conceptual misunderstandings or providing information on ethical issues. Synthetic biologists themselves can also clear up confusion. |

| **Author, Year** | **Ethical Debate** | **Key Concepts** | **Conclusions and/or Recommendations** |
| --- | --- | --- | --- |
| Smith, 2013 | The potential benefits of synthetic biology applications, particularly in the medical field, are apparent; however, as the field is still in its infancy, it is impossible to predict these benefits. Issues such as iatrogenesis and bioterrorism are possible adverse outcomes of synthetic biology.  The precautionary approach to synthetic biology poses several problems in the context of positive or negative outcomes. The main problem is the need for more knowledge associated with synthetic biology.  From a utilitarian perspective, the laissez-faire argument can be used to give synthetic biology a wide berth.  While no moral theory is perfect, the utilitarian argument in synthetic biology is internally consistent and superior in its application. | Utilitarianism  Precautionary principle  Laissez-faire | The benefits of synthetic biology studies and the knowledge they generate are expected to be considerable, especially in fields such as medicine. In this respect, adherence to the precautionary principle in terms of following safety rules in regular scientific and microbiological studies is sufficient to prevent many negative consequences, and there is no need for excesses. |

| **Author, Year** | **Ethical Debate** | **Key Concepts** | **Conclusions and/or Recommendations** |
| --- | --- | --- | --- |
| Kaebnick  et al., 2014 | An emerging technology is viewed positively by society, provided that it is monitored and its possible downsides are taken into account; it is even expected to encourage the development of this technology. Ethical debates on synthetic biology are conducted along this axis.  One of the critical issues is that this dynamically developing field should not feel complacent; it should always recognise that there is a field of uncertainty and moderation. The field contains both practical and conceptual ambiguities.  Three main concerns arise about synthetic biology: (1) the intrinsic value of synthetic biology, (2) concerns about the concrete benefits and harms of synthetic biology, and (3) concerns about justice. | Human-nature relationship  Potential benefits and harms of synthetic biology  Fair distribution  Deliberative approach | Making a final judgement on the ethical concerns associated with synthetic biology is impossible.  It is possible to apply a multidimensional methodology to the process to address potential issues that may arise.  In this context, public consultation is one of the most fundamental steps. While the public should be informed appropriately, their input should also be sought.  It is also important to be well-informed about the concepts of synthetic biology, to carry out ethical analyses on an ongoing basis, and for professionals to self-evaluate their actions.  Information ethics should be given importance; attention should be paid to using information for practical purposes.  Frequent analysis of values and outcomes is also important. |

| **Author, Year** | **Ethical Debate** | **Key Concepts** | **Conclusions and/or Recommendations** |
| --- | --- | --- | --- |
| Raho, 2014 | The first set of ethical issues is the physical harm associated with synthetic biology’s current and foreseeable risks. These are issues of biosafety and biosecurity. Biosafety concerns the release of organisms into the environment. The unintentional release of these organisms into nature poses significant threats to agriculture, the environment and public health. Synthetic biology has the potential to produce, replicate and modify dangerous viruses or bacteria.  Intrinsic objections to synthetic biology, which include issues ranging from metaphysical views to concerns about the products of creation, form the second group. These can be expressed as playing God, blurring traditionally accepted distinctions and going against nature. According to metaphysical and theological understandings, the playing God argument does not specify how humans should intervene in creation. Trying to respect creation in different ways means paying attention to the fundamental aims of science. The epistemological version of the same argument is mainly disingenuous. Scientists aim to promote the responsible use of synthetic biology products. The biosafety concern is not inherent to synthetic biology; it is an empirical objection.  The motivation behind the idea that synthetic biology blurs the traditionally accepted distinctions between organism and machine, and animate and inanimate, is to downplay the moral status of a new organism once it has been created. If these organisms have intrinsic value, then direct obligations towards them must be considered. | Physical harm  Biosafety  Biosecurity  Playing God  Intrinsic value | Synthetic biology represents a potentially radical change in our relationship with nature. An important question in this early and manipulative field is how scientists will react when they are able to create synthetic cells from scratch.  While internal objections to synthetic biology are forcing us to rethink our relationship with nature, they are not enough to stop research. |
| **Author, Year** | **Ethical Debate** | **Key Concepts** | **Conclusions and/or Recommendations** |
| Baertschi, 2015 | Grounding life, mind, personality and subjectivity in non-natural or non-physical explanations is very difficult. With, or thanks to, modern science, a more solid metaphysical ground is being established.  If life is having come into being in some way and having certain characteristics, then beings with vital functions can be regarded as life forms or as having life. Accordingly, life can exist. Although some scientists deny it, creating beings with vital functions from synthetic biology products is a creation. With such an acceptance, the metaphysical view of existence may also change.  A connection and parallel between neuroscience and synthetic biology, scientific data and metaphysical concepts can be drawn. | Nature of life  Free will  Moral status  Determinism | The mind is strongly immanent in the body. It is difficult to argue that the mind or psychic function is independent; experience and metaphysics converge.  Developments in synthetic biology and neuroscience fields may change many metaphysical assumptions and arguments. In this context, many concepts such as life, mind, personhood and subjectivity become more challenging to explain or attribute to unnatural means. |

| **Author, Year** | **Ethical Debate** | **Key Concepts** | **Conclusions and/or Recommendations** |
| --- | --- | --- | --- |
| Chen et al., 2015 | There is a widespread view that synthetic biology, which aims to design and build new biological functions and systems not found in nature, will redefine the boundaries between natural and artificial.  Deontologically, long-standing debates continue about the right to play God, intervene in future living beings, and human intervention in natural development.  With the introduction of artificial life, the debate between conservatives and radical Darwinists over whether God alone (or nature) is capable of creating everything has resurfaced.  Synthetic biology also raises biosafety, biosecurity, stakeholder engagement and intellectual property rights issues. | Playing God  Natural/artificial distinction  Biosafety  Biosecurity | There is no reason to negate all the achievements of synthetic biology because of moral, ethical and religious debates.  Given the religious and ethical debates in many disciplines and the goal of synthetic biology research to benefit humanity, it seems unnecessary to hold researchers to overly strict moral standards.  Respect for life does not mean modifying any life form on earth is inappropriate. |

| **Author, Year** | **Ethical Debate** | **Key Concepts** | **Conclusions and/or Recommendations** |
| --- | --- | --- | --- |
| Magnin, 2015 | Modern technoscience shapes our relationship with nature, the world and ourselves. Among the many questions that arise at the intersection of the biological sciences and philosophy, with an economic and legal background, there are three main ethical issues: (1) Issues of biosafety in terms of balancing benefits and risks: What happens if newly created microorganisms are released into nature and mutate? In the context of terrorist threats, how should biosecurity be addressed if new and dangerous viruses are created? (2) When this technology is applied to humans, to what point and at what cost can human limitations be overcome? Is the aim to humanize or to escape human finitude and deny human death and destiny? (3) Synthetic biology is changing the relationship between living things and life, affecting our view of what is natural and what is artificial, what is animate and inanimate.  The robustness of microorganisms produced by synthetic biology raises the issue of fragility, a term used for all living things. Robustness refers to the insensitivity of a biological system to physical and chemical changes in its environment. Vulnerability is an inherent and ontological characteristic of all living things, although the degree of vulnerability varies significantly from one living thing to another. In this way, complete equality is achieved. The idea of vulnerability in all living beings is an appropriate starting point for studying ethical behavior in the new era of biotechnology. | Vulnerability  Robustness | In the context of the opportunities offered by synthetic biology and other technosciences, with their benefits and risks, being responsible for the living beings of today and tomorrow means thinking about their complex and fragile nature. Today’s technological power demands more than ever that we act responsibly in the face of the fragility of living beings.  Ethics does not prohibit or hinder scientific and technological development. Still, it defines what helps human beings become more efficient in modern technology that respects living beings and life. In this context, philosophers and scientists should work together. |
| **Author, Year** | **Ethical Debate** | **Key Concepts** | **Conclusions and/or Recommendations** |
| Newson, 2015 | The potential applications of synthetic biology raise many philosophical and ethical questions, such as (i) What characteristics must a being have to be defined as alive? (ii) Can synthetic biology research that creates new living biological organisms be justified? (iii) Does it matter how a being becomes alive? (iv) When a living being is created, at what point should it have the rights and the right to life attributed to those with moral status?  Creating new life forms also reveals intrinsic biological knowledge that is valuable in itself. This can inspire admiration for the complexity of life rather than seeing it as mechanical. Risk is not enough to condemn the creation of new life forms as long as responsible and well-assessed risk management exists. It is important to ensure that synthetic biology products are not in the wrong hands, harming populations or the environment.  An essential ethical justification for biosafety measures is protection from harm. It is important to ensure that synthetic biology products do not harm populations or the environment in a bad situation.  Biosecurity issues are related to dual use. This describes how the same research can be used for good and bad purposes.  Benefit sharing involves ethical issues such as whether it is appropriate to patent an artificially synthesized genome. The role of patents and other intellectual property issues in influencing the price and availability of synthetic biology products also remains essential. | Intrinsic value  Biosafety  Biosecurity  Dual use  Benefit sharing | It is clear that synthetic biology does not raise entirely new ethical issues and that ethical analysis within synthetic biology should not be defined as a separate research area within bioethics. However, ethical questions such as the best governance strategy or the appropriateness of ethics of knowledge have yet to be resolved for synthetic biology or emerging technologies.  The field could benefit from an approach to ethical analysis that can both think broadly about possible future scenarios for synthetic biology and focus on some of the more specific implications that are possible or real.  Continued critical reflection on the methodology of bioethics in synthetic biology will also enable critical reflection on the methodology of bioethics more generally. Given the field’s interdisciplinary nature, it may also lead to some new observations. |
| **Author, Year** | **Ethical Debate** | **Key Concepts** | **Conclusions and/or Recommendations** |
| Wareham  et al., 2015 | The precautionary principle is seen as an epistemic requirement to identify the conditions under which it is dangerous to carry out a research project. The precautionary principle is sensitive to the degree of potential harm in the circumstances of its implementation, and the degree of potential harm that an activity may cause should be above the required standards.  Synthetic biology carries the risk of catastrophic consequences along with the potential benefits. If the potential harm of an activity is more significant, weaker evidence that harm will occur is required to prevent it. This principle is the rule of evidence-harm proportionality embodied in a risk prevention framework.  Risk is modelled as the expected value of an undesirable outcome. This value is not the expected value of an event in the usual sense but a way of combining probabilities and outcome values for comparison and decision-making.  The evidence-harm proportionality rule recognizes that if the potential harm of an activity is more significant, there must be a greater probability that no harm will occur for the activity to occur. If the potential harm of an activity is greater, the probability of harm must be lower for the activity to be prevented. A threshold value indicates that potential harm can be ignored below a certain level of evidence. If harm is not sufficiently likely, it does not need to be considered. Accordingly, a synthetic biology project will escape an injunction if the likelihood of harm is below a specified minimum probability threshold. | Precautionary principle  Evidence-harm proportionality rule  Threshold value  Objective Bayesianism | The threshold value, which sets a minimum level, should be endorsed by society and reflect society’s informed judgement of the probabilities that can and should be ignored. They should be ignored if harms are unlikely to fall below the threshold.  Willingness to pay is a common way of assessing people’s preferences for risk. Using this method, the researcher can ask how much people are willing to pay to avoid low-probability events and derive a threshold. If a synthetic biology project has a certain probability of harm, this predetermined probability can be compared to the threshold value and action taken (or not) consistently.  Even if the epistemic benefits of community-determined thresholds are rejected, there are ethical benefits. Deliberative threshold setting ensures respect for people’s autonomous preferences by involving more stakeholders in decisions about the level of risk that directly affects society. In this way, it increases the legitimacy of decisions about the potentially dangerous consequences of synthetic biology projects.  Deliberative thresholding provides a reasonable way of describing the probability space considered when making decisions about synthetic biology projects. The objective Bayes rule is an appropriate tool for reasoning about decisions in the dark when dealing with synthetic biology projects and for assessing threats above a threshold value. |
| **Author, Year** | **Ethical Debate** | **Key Concepts** | **Conclusions and/or Recommendations** |
| Gómez-Tatay et al. 2016 | Ontological personalism, an anthropological theory that defends the objective value of human beings based on their ontological structure, can be used to provide an ethical framework for synthetic biology. The principles the preservation of life and genetic identity, the principle of healing, the protection of the ecosystem and the environment, ontological and axiological differences between humans and other living beings, and community competence, can be adapted to synthetic biology. Meaning of life, moral status, biosafety, biosecurity, synthetic humans and social awareness can be based on these principles.  Direct (medical applications) or indirect (release of synthetic biology products into the environment) applications must ensure that the integrity and dignity of human life are not violated. Despite the potential benefits, the possibility of misuse raises several dangerous risks that doubt the wisdom of acquiring and disseminating knowledge in this field.  The moral status of an organism is given by the properties that determine the consequences of its actions. The ontological and axiological differences between humans and other living beings result from human-specific characteristics.  The risks of releasing synthetic organisms, advances in safety systems (physical measures, limiting the survival of organisms to certain conditions), biosecurity such as the DIY movement and the risk of misuse, advances in security systems, etc. will also allow participation in the debate and help formulate appropriate regulations. | Ontological personalism  Meaning of life  Moral status  Biosafety  Biosecurity  Synthetic humans  Social awareness | The terms design, construct or recreate are more appropriate than create when discussing what is being done in synthetic biology, and their use will avoid concerns.  In addition to its potential benefits, synthetic biology poses real risks to human health and the environment. Interactions between synthetic biology products and other organisms and the environment may occur with unknown consequences. Therefore, biosafety measures should be developed for new developments and applications in this field. In addition, possible misuse of synthetic biology, such as the production of biological weapons or the design of synthetic organisms or new pathogens to produce toxins, is also a risk to be considered.  Biosecurity problems should be avoided by imposing restrictions on research and the dissemination of information, legislative control over the market for genetic material, and establishing some bureaucracy to oversee the DIY movement.  An educational initiative involving the synthetic biology community, the public and legislators is needed to promote a properly informed debate that can create the necessary regulations to ensure the safe, appropriate and fair development of synthetic biology. |
| **Author, Year** | **Ethical Debate** | **Key Concepts** | **Conclusions and/or Recommendations** |
| Gregorowius et al., 2016 | Responsible research and innovation (RRI) is the latest model for addressing risk and societal or ethical issues. It aims to move from assessing outcomes and impacts after a technology has been developed to engaging in the innovation process as the technology is being developed.  Evaluating and developing emerging technologies and applications should include ethical thinking and foresight. Including these issues should help analyze, interpret, and shape technological applications and their potential impacts on society and the environment.  Following the synthetic production of the first viral and bacterial genomes, synthetic biology has been discussed in the context of bioterrorism, biosecurity, laboratory safety, environmental protection, intellectual property rights, and general ethical issues from the perspective of technology assessment.  With the shift from ELSI/ELSA models to the RRI approach, integrating societal and ethical concerns in synthetic biology into innovations to address future challenges has also come to the fore.  The challenges of applying the responsible research and innovation model to synthetic biology include (1) uncertainty about the possibilities of this technoscience, (2) finding appropriate models for engaging the community, and (3) influencing the innovation process without inhibiting scientific curiosity and engineering creativity. | Responsible research and innovation | Responsible research and innovation emphasize that assessing an emerging technology such as synthetic biology should not focus solely on concerns about its risks and negative impacts. Instead, technology assessment should be embedded in innovation and invention. One of the main challenges in this process is engaging society and jointly analyzing the societal and ethical goals between natural and social scientists to define what constitutes responsible research and innovation.  Implementing the RRI model for synthetic biology must manage the balance between shaping research and innovation through community engagement and leaving enough space for scientific curiosity and engineering creativity. |

| **Author, Year** | **Ethical Debate** | **Key Concepts** | **Conclusions and/or Recommendations** |
| --- | --- | --- | --- |
| Hagen, 2016 | Societal debates about synthetic biology in science policy run the risk of preventing or reducing innovation. Several intermediate strategies have been developed to avoid these problems in science policy: attempts to address debates about the importance and legitimacy of relying on scientific evidence in regulatory processes; attempts to address the economic risks or limited scope of existing and additional regulatory science policy recommendations, emphasizing follow-up proposals; emphasis on self-assessment or self-management strategies; legitimizing the process by developing relationships with the humanities and social sciences and the arts, resulting in good scientific communication. Although these initiatives are not well established, they are primarily based on an “instrumentally realized motivation.” Such an initiative is not limited to the scientific community but is supported by policymakers and funders.  Synthetic biology is a dynamic technoscience and an important example of science governance and the widespread use of concepts such as responsible research and innovation. There are also developments in the ethical debates surrounding synthetic biology. | Responsible research and innovation  The danger of ethics and humanities becoming a means of legitimizing science policy | In synthetic biology, an emerging field, it is important and necessary to have ethical discussions with broad participation to ensure social credibility and to show that the right things are being done and mistakes are being avoided. Participation and collaboration prevent mistakes and increase legitimacy.  Synthetic biology is an area where economic and political expectations influence science policy. Both economic benefits and solutions to problems are expected. As a result, interest in the field is growing. Despite this growing interest, risks and difficulties should be addressed.  Several standardized technology assessment studies and initiatives have been established in this context, and the concepts of ELSA and RRI have come to the fore. Responsible research and innovation are associated with transparency, openness, interdisciplinarity, philosophy and the humanities. Another issue highlighted by RRI relates to economic growth and is essential for funding.  Synthetic biology research must consider tensions based on political and economic expectations. The primary strategy for synthetic biology is soft governance, including ELSA and RRI activities, with support for science and the involvement of an increasing number of stakeholders. In this context, assessments for future research should consider political and scientific aspects and tensions. |

| **Author, Year** | **Ethical Debate** | **Key Concepts** | **Conclusions and/or Recommendations** |
| --- | --- | --- | --- |
| Laird et al., 2016 | Scientific developments in synthetic biology are rapid and dynamic, and policy development is challenging. The responsible research and innovation model can be adapted to a rapidly developing but relatively unknown field such as synthetic biology. According to this approach, limited governance capacity, jurisdictional confusion, funding equity and lack of strategic approaches are significant shortcomings for synthetic biology.  Coordinated coexistence between the Convention on Biological Diversity and other policies provides an important method and basis for implementing RRI, especially for social justice, sustainability, biosecurity and other issues that may arise with emerging technologies. It can also be said that unethical practices will be reduced. | Responsible research and innovation | As synthetic biology and other new technologies develop, there is an urgent need to embed the responsible research and innovation model in an open and holistic view of all research processes, in line with other policies. Among other benefits, this will facilitate the prevention of unethical practices. |

| **Author, Year** | **Ethical Debate** | **Key Concepts** | **Conclusions and/or Recommendations** |
| --- | --- | --- | --- |
| Macnaghten et al., 2016 | Science has an intrinsic meaning of objectivity and reliability and an extrinsic meaning that considers social influences. In the Enlightenment, the positive attitude to science is utilitarianism, impartiality and objectivity.  In the 20th century, the relationship between technoscience and power changed, and the concept of responsibility came to the fore. Scientists need to think not only about external risks but also about the goals and motivations of science. In the case of synthetic biology, this is inevitable due to uncertainties and ethics.  Society is an important factor in synthetic biology research. If the social harms are high, the likelihood of rejection increases; if the benefits are high, acceptance increases. There should be science policies that consider social values in the scientific process. In this context, the responsible innovation model comes to the fore. According to this model, a general assessment of foreseeable factors can be made when discussing the social and ethical aspects of an emerging field such as synthetic biology. | Responsible innovation | Institutional and governance changes are important.  The most important condition for responsible innovation to be valuable and sustainable is that governments operationalize it with broad participation for a wide range of public goods.  If the responsible innovation model is not institutionally and genuinely supported, it may be limited to instrumental use over time. |

| **Author, Year** | **Ethical Debate** | **Key Concepts** | **Conclusions and/or Recommendations** |
| --- | --- | --- | --- |
| Schmidt et al., 2016 | Understanding the limitations of synthetic biology as a future-oriented technoscience is important for addressing potential risks. Prospective technology assessment provides an early opportunity to analyze, understand, anticipate, and, where appropriate, shape this emerging new technology.  Hans Jonas’s ethics and the imperative of ethics in prospective technology assessment can provide a basis for evaluating concepts of responsibility in synthetic biology.  The dynamic nature of synthetic biology makes it difficult to control. This inevitably creates uncertainty, and what humans produce becomes inaccessible and uncontrollable. This changes the notion of rational design about technology.  In Jonas’s report on new technologies, the ethical approach is precautionary. Actions must be an existential imperative responsibility for human beings. Imperative responsibility is “acting in such a way that the effects of the action do not harm a life like the present one in the future”.  Recognizing the future importance of biology, Jonas said that the possibilities for control over technology will diminish. This situation requires ethical thinking that includes future predictions and assessments. In this context, the design of safer and more controllable systems should be considered from the beginning of synthetic biology. It is also important that the field is sustainable. These factors can be addressed through technology assessment processes. | Prospective technology assessment  Precautionary principle  Imperative of responsibility | From an ethical point of view, it is a fundamental task to take up a late-modern technology such as synthetic biology, which involves uncertainties, and to develop procedures to address, limit, shape or deal with the problems that will arise. In this respect, Hans Jonas can be seen as a pioneer. His future-oriented ethical perspective is valuable for understanding the problems.  Prospective technology assessment can provide an interdisciplinary, critical-reflexive analysis and evaluation of the technoscientific nature of the emerging technology wave. |
| **Author, Year** | **Ethical Debate** | **Key Concepts** | **Conclusions and/or Recommendations** |
| Ahteensuu, 2017 | Ethical concerns can be divided into two groups: intrinsic and extrinsic concerns. Intrinsic concerns embody the idea that research and practical applications of synthetic biology are morally questionable because of some features of the technology (its use), regardless of the consequences. These include the question, “Does the creation of new life forms cross the moral boundaries of playing God, unnaturalness or human hubris?”  According to extrinsic concerns, research and practical applications of synthetic biology may be morally questionable because of their known, predicted or possible consequences. “Does building new kinds of organisms and species change how we perceive nature and ourselves? Alternatively, does it lead to a misjudgment of the status of synthetic organisms?” These concerns are based on the slippery slope argument. “Does the use of synthetic biology in the form of expensive treatments available only to a privileged minority at the expense of the general health care of the majority lead to an unfair distribution in society?”  Extrinsic concerns include potential adverse effects on human health, animals and the environment. Biosafety and biosecurity are prominent in this context. Biosafety refers to principles, practices, and specific measures to prevent unintended and unexpected consequences. Biosecurity refers to principles, practices and specific measures to prevent the misuse of synthetic biology. Such risks form a continuum from the most straightforward bioterrorism concerns to biological warfare. | Intrinsic concerns  Extrinsic concerns  Biosafety  Biosecurity  Precautionary principle | A common response to biosecurity threats is to apply the precautionary principle. However, another question is what precautionary measures can be justified in the face of these risks.  It is generally accepted that precautionary measures can be bans or phaseouts, moratoria, premarket testing, labelling and requests for extra scientific information before proceeding.  Another type of precautionary response could be the establishment of new precautionary risk assessment methodologies. In this case, the focus is not only on dealing with identified threats but also on the methods to anticipate and assess threats in the first place. |
| **Author, Year** | **Ethical Debate** | **Key Concepts** | **Conclusions and/or Recommendations** |
| Häyry, 2017 | As an academic discipline, the ethics of synthetic biology examines questions of values and norms raised by synthetic biology as both a science and a branch of engineering. As a policy practice, it regulates synthetic biology research and production to prevent harm, reduce risk and manage conflicts of interest.  There are three concerns about synthetic biology: (1) that creating new life forms is either intrinsically bad or leads to bad consequences; (2) that unwarranted fear or unfounded optimism guide work or regulation in the field; (3) that engineering life, like all emerging technologies, needs public approval to proceed smoothly.  Creating new life forms may produce something physically dangerous, or its implementation may be socially harmful. Creating new life forms may also be wrong on symbolic grounds. Traditional symbolic arguments criticised in this context are unnaturalness and playing God. Other symbolic objections to introducing new life forms into the natural environment concern purity (in some undefined sense) and the polluting effect of human-made entities. | Precautionary principle  Hopeful principle | The precautionary principle is a common standard in environmental policy and technology assessment. It says that if the consequences of a decision could be catastrophic, the decision should only be taken once there is sufficient scientific evidence that it is safe. Critics of the precautionary principle point out that the mere fact that someone questions safety, for whatever reason, could lead to the banning of the most beneficial practices.  Almost all new advances in science and technology, including synthetic biology, are unpredictable because they have yet to be tried. In this context, the precautionary principle is not a good policy. It does not lead to reasonable caution but to unwarranted fear.  The hopeful principle is based on the belief that technological progress is essential to the future well-being of humanity and that obstacles based on fear and anxiety cause more suffering than even the most dangerous inventions. Because it can lead to beneficial advances, synthetic biology can proceed unhindered, under ethical oversight, unless it can be reliably shown to be unsafe. |

| **Author, Year** | **Ethical Debate** | **Key Concepts** | **Conclusions and/or Recommendations** |
| --- | --- | --- | --- |
| Heavy, 2017 | Applications of synthetic biology have positive impacts on agriculture, medicine, fuel production and the advancement of science, but also potential dangers. This raises issues of biosafety and biosecurity. Synthetic organisms could be released into the environment and affect the evolutionary process of other organisms with which they interact.  Synthetic biology raises the possibility of taking biological weapons to a new level. All research, even the most beneficial, raises the issue of dual use, i.e. the possibility of biological weapons being made available to the public.  The unknown knowns of synthetic biology are that the dangers are great. The potential benefits are huge and, at best, revolutionary for humanity, which is why research continues despite the obvious potential risks. The dual use nature of synthetic biology means that even the most beneficial developments have the potential to be used negatively. Ethically, it differs from other areas of science and technology in that the potential for both benefit and harm are much greater. In the worst-case scenario of synthetic biology, denial and willful ignorance of ethical considerations could lead to the most significant disasters humanity has ever experienced. | Consequentialism  Biosafety  Biosecurity  Dual use | Synthetic biology offers both great potential benefits and great potential dangers. Synthetic biology research is unethical and cannot be supported from a consequentialist perspective unless the risks are minimized.  Weighing the potential benefits against the potential harms, the consequentialist approach will not support synthetic biology. However, this may change if regulations can be developed that allow the beneficial side of the research to flourish while minimizing dangerous practices. Appropriate field regulation is an ethical imperative and should go beyond sanctions. Regulation should ensure that the likelihood of worst-case scenarios occurring is minimal.  A consequentialist analysis is invaluable in identifying synthetic biology’s potential benefits and dangers and guiding ethicists and lawmakers on how to respond in the short term. However, it is not relevant to determine whether synthetic biology is ultimately ethical and whether humanity should engage in this endeavor.  Consequentialism fails when outcomes can only be meaningfully predicted in the short term. |

| **Author, Year** | **Ethical Debate** | **Key Concepts** | **Conclusions and/or Recommendations** |
| --- | --- | --- | --- |
| Holm, 2017 | The malicious biohacking movement is at the center of the debate about synthetic biology, which is allegedly engaged in do-it-yourself (DIY) synthetic biology. This movement has considerable powers, described in the literature as being able to design and synthesize the DNA or RNA of the new pathogen, culture it in the quantities required, make it into an aerosol or powdered weapon, package it and disseminate it. However, all these powers are mythical because they are not based on any meaningful real-world application.  The pragmatic role of the bioterrorism argument is that the constant development of new technologies to which it can be applied allows middle-aged or older bioethicists to repeat the arguments.  Nevertheless, there are some concerns about the use of the bioterrorism argument. Two philosophical problems arise (1) the difficulty of accurately assessing the comparative importance of analyzing different philosophical questions; (2) even if comparative importance can be determined, the fallacy of ignoring the importance of a particular question and the importance of a particular person’s thinking about that question.  The question of bioterrorism allegedly created by synthetic biology does not seem important in the first sense. Analyzing or solving this question will not lead to a significant philosophical advance, considering the problem of dual use in general. | Bioterrorism | The argument serves several functions that depend on something other than being valid and sound but only on being rhetorically persuasive. The use of argument in bioethics is very similar to its general use: to establish synthetic biology as an important field of activity.  Proponents of synthetic biology paradoxically use this argument to demonstrate the power and utility of the new technology they are investing in, and politicians use it to defend illiberal security and surveillance policies.  The issue of bioterrorism is not as important as the literature suggests. Bioethicists talk more about systemic injustice or abuse of power than about bioterrorism. |

| **Author, Year** | **Ethical Debate** | **Key Concepts** | **Conclusions and/or Recommendations** |
| --- | --- | --- | --- |
| Pang et al., 2017 | The ethics of synthetic biology is practical and describes the moral issues arising from experimentation and research. The role of practical ethics in synthetic biology is to encourage the maximization of the benefits of this innovative technology and the minimization of the harms and risks that are by-products of the technology. This ethical stance generates two types of response: a tendency to create regulation to encourage an appropriate ethical response or an insistence on developing the technology to maximize benefits without regulation.  The ethics of dealing with living organisms, including the engineered organisms of synthetic biology, are not only norms and principles but also an attitude of responsibility and respect.  It could be argued that synthetic biology is a kind of hubris to manipulate cells for humans, sometimes called playing God. The critical question about synthetic biology is not who created it but what attitude and mindset is behind the science. This ethical perspective questions both the creation and the attitude and perception, as opposed to the risk and safety or experimental perspective and the life and nature perspective.  The ethical assessment of research affects researchers’ attitudes and the management of R&D through generation, sharing and exchange. Research ethics shapes society’s perception of researchers and builds public trust and respect for them. In this sense, the ethical nature of public policy is not about living or non-living things but about human attitudes and behavior towards them. | Practical ethics  Playing God | ---- |
| **Author, Year** | **Ethical Debate** | **Key Concepts** | **Conclusions and/or Recommendations** |
| Takala, 2017 | Much of the ethical debate on synthetic biology concerns its potential consequences.  Biosafety and biosecurity issues are critical to mitigating the risks of synthetic biology. The harms, if any, are more likely to be seen before the real benefits are realized.  In order to design and construct biological parts, devices and systems to perform specific useful functions, and to design and construct biological parts, devices and systems for this purpose, it is necessary to know more about the function and interaction of genes, biological systems and the environment. However, not much knowledge is needed to create unpredictable, unstable organisms. Synthetic organisms that are accidentally, carelessly or maliciously released into the environment are the greatest threat that synthetic biology currently poses.  The shift from manipulation to creation potentially undermines the value of life. The idea of looking at nature as a void to be filled with whatever we want threatens the tendency to see life as something valuable. If the question is, “Does life have value in itself, or is there something valuable in life?” it means that there is something valuable. Synthetic biology cannot necessarily threaten that value. However, synthetic biology creates a grey area for defining a life with moral value. | Biosecurity  Biosafety  Value of life | As the consequences of synthetic biology are mainly unknown, more emphasis should be placed on biosafety and biosecurity.  Without any theological implications, it could be said that synthetic biologists are playing God by creating new life forms. However, this argument oversteps the boundaries of what is acceptable and is dangerous in the wrong hands.  In this context, synthetic biology professionals should consider all possible consequences before moving forward in this area. |

| **Author, Year** | **Ethical Debate** | **Key Concepts** | **Conclusions and/or Recommendations** |
| --- | --- | --- | --- |
| van de Poel  et al., 2017 | Safe-by-design aims to ensure that safety issues are considered and discussed throughout new technologies’ R&D and design phases. Issues such as releasing synthetic organisms into the environment have highlighted the importance of safe-by-design. The safe-by-design approach provides a framework for risk analysis, management, and safety once the technology is implemented. However, it is essential to ensure real safety and the possibility of avoiding risk altogether. In this context, there is uncertainty in human behavior. It is better to accept this uncertainty and use it as a potential source of safety than to design for complete security.  Rather than designing for safety, it makes more sense to focus on the responsibility for safety. Designers should consider where the responsibility lies and design technologies accordingly.  Designing in uncertainty can be a way of taking responsibility for the safety of their designs and protecting them from harm. There should be a shared responsibility in these processes. However, responsibility should also be contextualized.  For example, in the case of dual use, while the intentions may be good, the arising problems may outweigh the responsibilities. Uncertainty in research should, therefore, be accepted, but responsibility should be addressed. This kind of responsibility is also important for technology owners; technology holds the potential for change and transformation in their hands, and with that comes a moral responsibility. A sensitivity to minimizing or preventing harm should be imposed. | Safe-by-design  Shared responsibility | Some heuristics can be used to decide how to share and distribute responsibility for safe-by-design.  Especially in fields such as synthetic biology, safe design cannot eliminate all risks and uncertainties. Instead, design and research that accepts uncertainty and takes responsibility is rational. In addition, it should be recognized that only some things in research and design processes can be solved with safety responsibilities and that conflicts of values may be unavoidable. In some cases, access to one value may be at the expense of another.  While there is not always an optimal solution, recognizing both the diversity of values and the different forms that uncertainty can take in design is critical to designing safe products that better meet a wide range of needs. |
| **Author, Year** | **Ethical Debate** | **Key Concepts** | **Conclusions and/or Recommendations** |
| Boldt, 2018 | The machine metaphor is central to a particular understanding of synthetic biology. It is important to express this understanding and shape the research accordingly. For example, it can help search for and identify the causes of intracellular processes, which in turn can help engineer the development of single-celled organisms with novel properties.  Synthetic biology uses the machine metaphor and its derivatives, the equivalent of information technology, as a central concept for describing, explaining and modifying intra- and intercellular molecular processes.  The widespread use of machine metaphors in synthetic biology can be seen as an expression of the ideals of rational design and construction being incorporated into biology and, thus, into life. The machine metaphor fits well into the big picture of what synthetic biology is and what it aims to achieve.  Synthetic biology’s place in scientific progress is closely linked to an ontological assumption. Explaining complex molecules means knowing the simple molecules contained in the complex structure, and explaining the function of an organism means identifying and analyzing the genetic structure of this organism. The function of a complex object is the result of the laws and regularities that govern the behavior of its parts. In this context, if the parts of an object and their functions are known, the behavior of that object can be reliably predicted. | Machine metaphor | The machine metaphor, a powerful concept in synthetic biology, can efficiently guide research to analyze the relationship between genetic parts and the organism’s whole functions and identify ways to design organisms. It systematically reduces characteristics of organisms, such as the evolutionary process and ecosystem interactions.  In terms of the machine metaphor, viewing a being as a moral agent or patient becomes questionable. From this perspective, the properties of living beings that justify the attribution of moral status should be reinterpreted as illusory phenomena since they are in tension with explanations regarding parts that produce specific, reliably predetermined effects.  The machine metaphor highlights certain aspects of the nature and potential of science and technology, and synthetic biology in particular. Inherent in this perspective is a tendency to overestimate the potential contribution of synthetic biology applications to solving societal and environmental problems. |
| **Author, Year** | **Ethical Debate** | **Key Concepts** | **Conclusions and/or Recommendations** |
| Chan, 2018 | Synthetic biology is likely to reveal transnational inconsistencies in the regulation of research and health care and variations in treatment. The expectation of profit as an incentive for research into synthetic biology therapies creates pressure to ensure a receptive market for these therapies. This pressure influences expectations, understanding and attitudes towards synthetic biology and encourages active commercialization in health applications.  The main driver of synthetic biology in health research is profit, which will influence users’ understanding of the underlying science, risks, outcomes and expectations of the success of treatments. Commercial pressures on the technology will change the relationships between developers, providers and users of synthetic biology therapies and the conditions under which people will choose to receive them. While synthetic biology therapies are in the testing phase, patients may wish to participate in trials of this potentially beneficial new treatment. This raises issues of clinical research and medical ethics, such as under what conditions should treatments still in the experimental phase be given or under what conditions should volunteers be allowed to participate in clinical trials. Decisions about experimental treatment involve not only patients/participants and clinicians/researchers but also policymakers who set the guidelines for what kind of research is allowed and ethics committees who interpret these guidelines. | Synthetic biology tourism  Commercializa-tion  Consumerism  Risk | To address the challenges posed by synthetic biology in healthcare, clinical research, medical practice, researchers/clinicians and participants/patients, a paradigm of science ethics is needed to support new ethical understandings of the role of patients and the relationship between science and society.  Global cooperation is needed to address research and medical tourism that may result from transnational regulations and research differences. An important factor in this is the mobilization of scientists and the international scientific community to set internal standards of conduct and to promote their procedures for upholding them.  Restrictions on access to commercialization and societal attitudes towards synthetic biology therapies should be carefully assessed, and mechanisms should be developed to ensure appropriate access to the technology (and discourage those who do not).  Global collaboration, access to technology, increased participation in research and strategies to promote openness should be developed.  As knowledge and practice are products of science, they should be accessible and used for the benefit of society. To make informed decisions about health technologies and to develop an understanding of science, access to information must be open. |

| **Author, Year** | **Ethical Debate** | **Key Concepts** | **Conclusions and/or Recommendations** |
| --- | --- | --- | --- |
| Stirling et al., 2018 | The complexity, diversity and scale of synthetic biology make it a unique field of research. This is also related to the dynamic and rapid changes in the field. The main ethical challenge in this context is to evaluate the pros and cons of options from different perspectives and justify the best course of action on behalf of society. Different approaches are needed in fields such as synthetic biology because there are many unexpected, multiple and not readily reducible problems.  For synthetic biology, in addition to cost-benefit analysis and the precautionary principle, the multi-criteria mapping method can be used to address problems. While this method will not solve every problem, it is a good example to illustrate the applicability of the precautionary principle in particular.  Unlike the probabilistic and reductionist approach to risk in cost-benefit analysis, the precautionary principle recognizes that uncertainties cannot be satisfactorily discounted. In this context, the precautionary principle assumes that regulatory assessment should be normative and assigns a crucial role to social values. This emphasizes that science is not unimportant, but that values and subjective frameworks should always be part of the analysis. Promising applications of the precautionary principle require consideration of democratic forms with multiple variables. | Cost-benefit analysis  Precautionary principle | Relying solely on cost-benefit analysis as the only method for evaluating technology is not enough. The precautionary principle and its applications can provide democratic approaches that are more inclusive and value-based.  More is needed to rely on cost-benefit analysis as the only way to evaluate technology. The precautionary principle and its applications can provide democratic approaches that are more inclusive and value-based. |

| **Author, Year** | **Ethical Debate** | **Key Concepts** | **Conclusions and/or Recommendations** |
| --- | --- | --- | --- |
| Funk et al., 2019 | Synthetic biology is a field intertwined with many discourses and metaphors about the meaning of life. It is a field in which new knowledge about life and new lives are created.  In ethical debates in synthetic biology, it is crucial to confront the concept of life. Determining the concept’s meaning is also crucial for action in the field.  Beyond popular and theoretical meanings, the concept of life has at least two concrete meanings that can be called burstwords. The first (burstword I) occurs when the meaning is unclear, and concepts such as life are not used appropriately. In these cases, the meaning is differentiated. In this definition of life, the misuse of metaphorical discourses such as genetic information and living machines can be questioned.  With a cross-disciplinary meaning, burstword II has an important interdisciplinary function: the term life is taken from an ambiguous and famous ground to a more natural ground. Through the naturalisation of life and the methodological reduction in synthetic biology, the positive aspect of the term is revealed, and a more robust epistemic meaning is achieved.  In terms of information technology, life and the metaphor represent knowledge, and the term life coincides with the meaning of burstword II. The meaning of life, including these forms of social life/behaviour, is naturalised.  Burstword II includes arguments about avoiding ethical problems, ethical fundamentalism or extremism about the concept of life. | Meaning of life | Life is a dynamic concept that is used in many different ways. However, its explicit use in a technoscientific sense is important for communication and research in this field.  The ambiguities of the concept of life are ethical and may involve risks and dangers about other issues. In this context, confusion and uncertainty of meaning should be methodologically reduced.  Burstwords II should have a more specific meaning and content that allows it to be used in other areas. This concept has a more controllable use of life. It can include constructive and developmental metaphors. It can be an understanding that allows for innovation and paradigm shifts, understanding across disciplines.  All these considerations are also about thinking outside the box in discussions about synthetic biology and life and developing new ways of thinking. |
| **Author, Year** | **Ethical Debate** | **Key Concepts** | **Conclusions and/or Recommendations** |
| Gómez-Tatay et al., 2019 | Despite the different definitions of life, synthetic biology does not exclude any of the characteristics used to define life. Thus, synthetic biology products should be considered organisms, regardless of their natural or artificial origin and their design and production purpose. They can be called synthetic organisms.  The principle of protecting the ecosystem and the environment obliges us to protect biodiversity and the environment and not to interfere with the interests of non-human organisms unless necessary for some human interest (provided it is morally right). Therefore, the organism’s interests must be considered as they are. The idea of how much better this organism might have lived if it had not been synthetic is morally irrelevant in the personalist view.  The recognition of intrinsic subjective value, intrinsic objective value, and inherent worth for other living beings does not equate them with the moral values of humans, consistent with the distinction between moral value and moral status.  From a personalist perspective, discussing the difference between natural and synthetic value is unnecessary. According to the principle of ecosystem and environmental protection, it is imperative to protect the interests of each individual. | Definition of life | A distinction must be made between organisms and machines to determine whether synthetic entities have moral value. Based on their immanent purpose, it is concluded that these synthetic entities, which retain entities of self-formation, self-preservation, self-reproduction and self-restitution, are considered organisms, regardless of their origin and the purposes of their production.  Since all organisms have purposes, and all living things are inherently valuable in terms of personalism, their purposes should be considered.  The designs of synthetic organisms have no moral significance.  There is no moral duty to protect synthetic species because they are not part of the biodiversity to be protected.  Since human interests are superior to those of other organisms, the primacy of the individual must always be respected. |

| **Author, Year** | **Ethical Debate** | **Key Concepts** | **Conclusions and/or Recommendations** |
| --- | --- | --- | --- |
| Heidari Feidt et al., 2019 | Synthetic biology is a promising biomedical research field that attracts significant investment and is expected to benefit patients through its clinical applications. In this context, the translational aspect of synthetic biology research is remarkable.  The translation of this research into practice is clinically and ethically significant. However, the ethical and social implications of the applicability of synthetic biology should be addressed. These studies are important for the benefit of society and global health, and therefore, the gap between research and practice needs to be reduced.  Society, researchers, funders and regulators have a moral obligation to promote the viability of synthetic biology research while taking care to prevent harm. This is known as the translational imperative.  Critical challenges for research are applicability, funding, economic, academic, and institutional challenges, ethical approval and legal issues.  The critical issue in translational research is translating the process into practice. In this context, ethics provides sensitivity to the issues and a positive view of the development of such studies in terms of public and social benefit. In particular, from a utilitarian perspective, the ethical promotion of these studies can benefit society and humanity globally. This requires a balanced, ethical assessment of harms and risks. | Moral obligation  Translational imperative | Society has a moral obligation for researchers, funders and regulators to promote the viability of synthetic biology research while taking care to prevent harm. |

| **Author, Year** | **Ethical Debate** | **Key Concepts** | **Conclusions and/or Recommendations** |
| --- | --- | --- | --- |
| Holm, 2019 | Synthetic biology is a threshold technology, open to positive developments but also to unexpected consequences. In this context, acting with caution and prudence is necessary. Decision-making processes related to synthetic biology and its regulation are an essential topic of debate. Decisions or approvals include laboratory research, what can be released into the environment, what can be used in humans, the commercialization of synthetic biology products, access to information and technology, and their possible consequences.  Deciding in the dark means making decisions under uncertainty and risk. The precautionary principle should not be invoked in decision-making because it needs to capture important features of decision-making and virtuous action sufficiently. The precautionary principle tends to minimize or ignore the benefits of synthetic biology. This diminishes courage as a virtue. In this context, a virtue-based approach is proposed.  However, how a virtue-based approach to synthetic biology is to be understood in the context of regulation is still being determined. It also needs to be clarified how virtue ethics’ emphasis on individual characteristics would work in institutional decisions and regulations such as synthetic biology. Thus, there are more robust alternatives to the precautionary principle than the virtue approach. | Precautionary principle  Deciding in the dark  Virtue-based decision making | Rather than focusing on harm, information and proposed action or one aspect, overall consistency can be considered, and the process can be balanced with counterfactuals (such as overall societal benefits) rather than focusing only on harm. The precautionary principle does not only focus on negative consequences. It can also be applied differently in different areas.  Although the use of the precautionary principle in decision-making in synthetic biology has been criticized, and a virtue approach has been proposed, the virtue approach needs to be more specific and clarify what it means in synthetic biology.  It has also been argued that various applications of the precautionary principle are still a practical approach to regulation and decision-making in synthetic biology. Nevertheless, the precautionary principle is not entirely immune to criticism. |

| **Author, Year** | **Ethical Debate** | **Key Concepts** | **Conclusions and/or Recommendations** |
| --- | --- | --- | --- |
| Coyne, 2020 | In their self-organization and purposeful behavior, organisms have immanently teleological ends. Certain entities enable an organism to fulfil these ends; others hinder it. Thus, every organism has a subjective logical imperative of good and bad since something can be better or worse for it, according to the satisfaction of its ends or otherwise.  Although a synthetic organism exists because of man as an organism, once it is created, it exists for itself. While this organism performs specific functions by the purpose for which it was created, it will also exhibit purposeful behavior in its efforts to continue its existence as a living being. Synthetic biologists define the ultimate interests of these organisms, but they also have their proximate interests. Through their proximate interests, these entities have instrumental value and, thus, intrinsic value. What matters for the definition of intrinsic value is not whether an entity is natural or artificial but whether it is alive. | Virtue ethics  Teleology  Intrinsic value | An adequate philosophy of life is important for an axiological analysis of synthetic organisms. This suggests that synthetic organisms have an instrumental and extrinsic value and an intrinsic value that only living beings have.  Virtue ethics, focusing on the good life and society, can explain the moral significance of accepting an endowed life and pursuing perfection. This perspective suggests that synthetic biology is a prime candidate for collective renunciation. |
|  |  |  |  |
| Kotzé, 2020 | Synthetic biology applications can address super wicked problems such as climate change, biodiversity loss, and species extinction.  The concepts of interdependence and relationality, as emphasized in Christian ecotheology, can help in the ethical evaluation of synthetic biology and can be used as an attempt to respond to the super wicked problem of climate change and resulting species extinction. | Wicked problems  Christian ecotheology | Synthetic biology could solve super wicked problems, such as climate change and the resulting extinction of species and could lead to an initiative that worsens the problems. |

| **Author, Year** | **Ethical Debate** | **Key Concepts** | **Conclusions and/or Recommendations** |
| --- | --- | --- | --- |
| Rohregger  et al., 2020 | Synthetic biology has applications in many fields, from food technologies to the creation of new organisms, and brings with it hopes and risks. In particular, the benefits, risks and threats to life arising from the creation and manipulation of DNA that does not exist in nature are the subject of bioethics.  Future-oriented ethical considerations regarding synthetic biology or technology should not be left to governments or politicians alone; societies should also evaluate developments from an ethical perspective.  Without appropriate ethical considerations, technological advances, particularly in synthetic biology, may produce results that threaten future social development, environmental protection, human dignity and life in the biosphere. Positive expectations should not overshadow possible negative consequences. In this context, ethical considerations and guidance are a fundamental responsibility. At the same time, bioethics should be a bridge between technology and science for the continuity of human life.  Synthetic biology has a dual and complex context; the field’s promise also carries threats and risks. Responsibility and prudence can be guiding virtues in this complexity and uncertainty.  The first step is to abandon the dualistic Cartesian view to addressing these issues. For life and organisms cannot be reduced to dualism. Life involves unpredictability and variability. Therefore, it is impossible to predict everything, and there is always a risk for the future. | Risk  Responsibility  Prudence | Synthetic biology is not characterized by the inability to achieve but rather by power, pride, and ability to do. Because of synthetic biology’s technological power, its outcomes’ unpredictability, and the risks associated with power and pride, prudence and the virtue of caution should have an important place in the field. This responsibility should not be limited to individuals; it should be political and collective rather than individual. Therefore, in addition to the responsibility of the scientist (and those who fund research), the legislator must also take responsibility.  In innovation and development, common sense and prudence should be applied to each new situation, society should be involved, and processes should be as transparent as possible.  In this context, synthetic biology should be guided by certain principles, as set out in some texts (USA et al., etc.): Public beneficence; responsible administration; intellectual freedom and responsibility; democratic deliberation; the precautionary principle; traceability principle; step by step and case by case principles. Adding other principles to these principles ensures the protection of the common good and life: Non-maleficence to the public; justice and proportional distribution of risks; government responsibility; risk maximization (giving priority to maximizing risks and problems that may arise from accidents, unintended development of genetically engineered organisms, or the possibility of dual use); reversibility principle. |
| **Author, Year** | **Ethical Debate** | **Key Concepts** | **Conclusions and/or Recommendations** |
| Sandler, 2020 | Ethical issues related to the environmental impact of synthetic biology, genetic engineering and gene drives relate to benefits and risks, opportunity costs and whether decision-making processes can be used responsibly. If the technology is effective, the risks are acceptable and manageable; the benefits outweigh the costs, the opportunity costs are low, oversight is available and public support is adequate. Synthetic biology, genetic engineering and gene drives will sometimes be a well-reasoned means of achieving known conservation goals, such as eradicating invasive species and improving disease resistance.  The ethical analysis of powerful emerging technologies is incomplete when these technologies are recognized as both tools and potential new ways of life. This is the case with synthetic biology, genetic engineering and gene drives. These technologies have the potential to reinterpret notions of value, meaning, significance, perspectives, and the concept of conservation and are of interest to conservation ethics.  The use of synthetic biology to adapt to anthropogenic change is often associated with moral hazard and technological solutions to a problem. It can be seen as a belief that the effects of problems can be addressed technologically without addressing their underlying causes. | Conservation ethics | An instrumentalist view of the technology dominates the debate on the conservation uses of synthetic biology. The ethical debate has therefore focused on risks and benefits, opportunity costs, decision-making processes, public engagement and acceptance.  However, the perspective of technology as a way of life is also important in analyzing the social and ethical dimensions. Synthetic biology may represent a new form of conservation practice, mainly if used to regulate genetic change in wild populations deliberately. This raises questions about power, meaning, values and worldviews.  Its power to drive genetic change in wild populations differs significantly from other conservation approaches. It enables the remodeling of the biological world at the genomic level by beliefs about how organisms should be. This qualitative difference in the range of human agency and the risks and uncertainties involved requires carefulness and vigilance.  Comprehensive ethical analyses, including instrumentalist and lifestyle issues, have been largely absent from the discourse on conservation genetic engineering in general and related to specific projects. |

| **Author, Year** | **Ethical Debate** | **Key Concepts** | **Conclusions and/or Recommendations** |
| --- | --- | --- | --- |
| Vallero et al., 2020 | Emerging biotechnologies, including synthetic biology, pose risks to human health and the environment and benefits for environmental engineering. Uncertainties about whether and how these applications will occur are challenging and may have downstream effects in the future.  New risk assessment methods have come to the fore for new technologies and uncertainties. These include the benefit-cost ratio, the minimalist model, the reasonable-care model and the good works model. The minimalist model is basically about following published ethical codes and standards for professionals in the field. In this context, actions outside the standards are explained by the concept of malpractice. It is more focused on a retrospective view and problems. This model needs to be more helpful, especially for developing technologies, as there is constant change and dynamism in the field. The reasonable-care model is similar to the minimalist model but differs in that it applies the principles of reasonable precautions and care to engineering practice. Although the concept of responsibility is difficult to define objectively, this model suits engineering practices with high standards. A good works model is one in which ethical decision-making in specific situations goes beyond standards to ensure sound engineering practice, considering public health and social welfare. It is the incorporation of proactive and protective ethical measures into design processes. | Risk assessment  Emerging technologies and good engineering practice in synthetic biology  Ethical assessment in technology | Environmental engineers use emerging biotechnologies and synthetic biology applications to achieve positive results. However, these applications can have potentially negative consequences. Therefore, environmental engineers should know their ethical responsibilities in their applications. This requires considering potential harm, ecological and public health issues, and potential benefits.  Different ethical assessment models can be used as the application areas of synthetic biology and emerging biotechnologies are variable. In the case of emerging technologies, systematic and preventive approaches come to the fore rather than models that focus only on malpractice. Nevertheless, it is difficult to identify a universally applicable model in every situation.  Ethical assessment models should be incorporated into the technology development process as early as possible. This creates an awareness of ethical issues that were previously unthinkable and unforeseen. |
