# Supplementary material for: The view of synthetic biology in the field of ethics: a thematic systematic review
Source: Front Bioeng Biotechnol. 2024 May 28;12:1397796. doi: 10.3389/fbioe.2024.1397796 (PMC11165145; doi:10.3389/fbioe.2024.1397796)
Supplement: Supplementary file 2 [file DataSheet2.docx]

Supplementary Material

The View of Synthetic Biology in the Field of Ethics: A Thematic Systematic Review

Ayşe Kurtoğlu^1*^, Abdullah Yıldız^1^, Berna Arda^1^

*** Correspondence:** Ayşe Kurtoğlu: [akurtoglu@ankara.edu.tr](mailto:akurtoglu@ankara.edu.tr), [aysekurtoglu87@gmail.com](mailto:aysekurtoglu87@gmail.com)

Supplement 3. Some concepts used in the article

**Consequentialism:** Consequentialism is a moral philosophy that holds that the consequences of one’s conduct are the ultimate basis for any judgment about the rightness or wrongness of that conduct. A morally right act (or omission) will produce a good outcome or consequence. The most well-known version of consequentialism is utilitarianism, which asserts that actions are morally right if they work to increase overall happiness. The optimal action is the one that maximizes total utility, which is measured as happiness or pleasure minus suffering or pain. In contrast to other moral frameworks, consequentialism focuses on the consequences of an action rather than how it is carried out. The results of an action are of greater importance than the intentions behind it or the inherent nature of the action itself. This approach can lead to ethical conclusions that differ significantly from those of other moral frameworks (Hallgarth, 2012).

**Epistemology:** Epistemology, a branch of philosophy, is concerned with the theory of knowledge. It examines fundamental questions such as the origins of knowledge, the influence of experience in creating knowledge, and how reason and experience interact. Key issues traditionally explored include the nature and sources of knowledge, its limits, and the reliability of knowledge claims (Flew, 1979a). Furthermore, epistemology examines the relationship between knowledge and certainty, the potential for error, the risk of universal skepticism, and the impact of new ways of understanding the world on our knowledge (Blackburn, 2005a).

**Instrumental value:** The instrumental value of an entity is contingent upon its usefulness to humans. Frequently, simpler living things are valued only for their usefulness to us. If we value an organism primarily for this reason, we see it as something to be used for our purposes. The instrumental value of an object is defined as the extent to which an object can serve a purpose set by the individual who values it.

**Intrinsic value:** The intrinsic value of an object is its inherent worth, independent of external factors. This value is not contingent on the subjective perceptions of beings capable of valuation. It means that an object is valuable in and of itself, not because of its usefulness to humans.

**Kant’s categorical imperative:** The categorical imperative is one of the fundamental concepts of Kantian ethics. Kant attempted to identify a universal ground for moral propositions. In this context, the categorical imperative is a rule that instructs us to act in a way that could potentially become a universal law, meaning that everyone could engage in the same action without causing problems. Based on these universal features, all imperatives of duty (specific moral claims) can be derived from the categorical imperative, as this will inform us of the principle underlying all such duties. The most important feature of the categorical imperative is that it is based on human reason. The general formula of the categorical imperative is to act only according to those maxims that can be consistently willed as a universal law – something said to be impossible for maxims aimed merely at material ends (Ameriks, 2015; Banham et al., 2015). This formulation has been a significant influence on the development of the concept of the autonomous individual, with a particular emphasis on the value of the human being.

**Metaethics:** Metaethics is a branch of philosophy that examines ethical concepts’ nature, foundation, and meaning rather than addressing specific moral issues. It is concerned with understanding morality and the structure of moral reasoning. Unlike normative ethics, which provides guidelines on what one ought to do, metaethics is more abstract, dealing with the origins and definitions of ethical principles. It addresses questions such as what it means for something to be good, to say an action is right or wrong, and whether moral judgments are universal or culturally relative. Metaethics can be thought of as a second-order discipline in philosophy, as it does not prescribe specific actions but investigates the nature of ethics and moral reasoning. This includes examining the language used to describe morality, the psychological processes underlying moral judgments, and the ontology of moral values, including whether they are real and objective or subjective and constructivist (Fisher, 2011).

**Ontology:** Ontology is a branch of philosophy that concerns the study of being and existence. The term, derived from the Greek word for being, was coined in the 17th century to describe the metaphysical study of what exists. Ontology explores fundamental questions such as the nature of existence and the categorization structure of reality. This branch of philosophy distinguishes between real existence and appearance, examining how entities within different logical categories, such as physical objects, numbers, universals, and abstractions, can be considered to exist (Flew, 1979b; Blackburn, 2005b; Lowe, 2005).

**Practical ethics:** Practical ethics applies ethical theories to real-world issues, bridging the gap between moral theory and practical life issues such as decision-making. It emerged as a significant field in the late 20th century, influenced by social movements and societal changes, focusing on problems in medicine, business, and policy. Unlike theoretical ethics, which deals with moral principles abstractly, practical ethics addresses specific dilemmas using philosophical and empirical insights to guide actions. Practical ethics aims to achieve reflective equilibrium, which entails adjusting moral beliefs based on new evidence and arguments. This ensures that decisions are well-informed and relevant to everyday situations (LaFollette, 2005; Singer, 2011).

**Translational imperative:** The translational imperative is about applying scientific discoveries to improve public welfare and overcoming barriers like financial constraints, organizational inefficiencies, and ethical concerns. These issues must be addressed early in the research process to implement new technologies and treatments successfully. Ethical considerations are fundamental, demanding that developments meet ethical standards and enhance societal well-being. This includes ensuring equitable access to new therapies. The imperative also encourages collaboration among researchers, policymakers, and healthcare providers to expedite the journey from laboratory research to clinical and public application, thereby maximizing the societal benefits of new scientific knowledge (Heidari Feidt et al., 2019).

**Utilitarianism:** Utilitarianism is a moral approach that prioritizes the satisfaction of desires and pleasure as fundamental elements of human good. It views the morality of actions as entirely dependent on their consequences for human or sentient well-being. Originating in Britain in the late seventeenth century and classically formulated by philosophers like Bentham, Mill, and Sidgwick, utilitarianism has discarded religious and social conventions to evaluate all moral questions by the standard of human happiness. Utilitarianism often encompasses a broader concept of well-being than mere pleasure in contemporary discussions. This encompasses anything that fulfills desires or contributes to happiness. This philosophy also adopts a form of outcome utilitarianism, asserting that the rightness of actions, motives, or political institutions is based solely on the resulting state of affairs’ impact on overall well-being (Slote, 2005).

# References

Ameriks, K. (2015). Kant. *The Cambridge Dictionary of Philosophy*, 555.

Banham, G., Schulting, D., and Hems, N. eds. (2015). *The Bloomsbury Companion to Kant*., 2nd edition. London: Bloomsbury Academic.

Blackburn, S. (2005a). Epistemology. *The Oxford Dictionary of Philosophy*, 118.

Blackburn, S. (2005b). Ontology. *The Oxford Dictionary of Philosophy*, 261.

Fisher, A. (2011). “Introduction,” in *Metaethics: An Introduction*, (Durham: Acumen Publishing), 1–9.

Flew, A. (1979a). Epistemology. *A Dictionary of Philosophy*, 101–102.

Flew, A. (1979b). Ontology. *A Dictionary of Philosophy*, 238.

Hallgarth, M. W. (2012). Consequentialism and Deontology. *Encyclopedia of Applied Ethics* Volume 1, 602–613.

Heidari Feidt, R., Ienca, M., Elger, B. S., and Folcher, M. (2019). Synthetic Biology and the Translational Imperative. *Sci Eng Ethics* 25, 33–52. doi: 10.1007/s11948-017-0011-3

LaFollette, H. (2005). “Introduction,” in *The Oxford Handbook of Practical Ethics*, ed. H. LaFollette (Oxford: Oxford University Press), 1–11.

Lowe, E. J. (2005). Ontology. *The Oxford Companion to Philosophy*, 670–671.

Singer, P. (2011). *Practical Ethics*., 3rd edition. Cambridge: Cambridge University Press.

Slote, M. (2005). Utilitarianism. *The Oxford Companion to Philosophy*, 936–939.
