# Supplementary material for: The view of synthetic biology in the field of ethics: a thematic systematic review
Source: Front Bioeng Biotechnol. 2024 May 28;12:1397796. doi: 10.3389/fbioe.2024.1397796 (PMC11165145; doi:10.3389/fbioe.2024.1397796)
Supplement: Supplementary file 3 [file DataSheet1.docx]

Supplementary Material

The View of Synthetic Biology in the Field of Ethics: A Thematic Systematic Review

Ayşe Kurtoğlu^1*^, Abdullah Yıldız^1^, Berna Arda^1^

*** Correspondence:** Ayşe Kurtoğlu: [akurtoglu@ankara.edu.tr](mailto:akurtoglu@ankara.edu.tr), [aysekurtoglu87@gmail.com](mailto:aysekurtoglu87@gmail.com)

Supplement 2. Included publications

Ahteensuu, M. (2017). Synthetic biology, genome editing, and the risk of bioterrorism. *Sci Eng Ethics* 23, 1541–1561. doi: 10.1007/s11948-016-9868-9

Baertschi, B. (2015). The Metaphysical Lessons of Synthetic Biology and Neuroscience. *C R Biol* 338, 617–626. doi: 10.1016/j.crvi.2015.06.002

Bedau, M. A., Parke, E. C., Tangen, U., and Hantsche-Tangen, B. (2009). Social and ethical checkpoints for bottom-up synthetic biology, or protocells. *Syst Synth Biol* 3, 65–75. doi: 10.1007/s11693-009-9039-2

Bensaude Vincent, B. (2013). Ethical perspectives on synthetic biology. *Biol Theory* 8, 368–375. doi: 10.1007/s13752-013-0137-8

Bhutkar, A. (2005). Synthetic biology: Navigating the challenges ahead. *Journal of BioLaw and Business* 8, 19–29.

Boldt, J. (2013a). Do we have a moral obligation to synthesize organisms to ıncrease biodiversity? On kinship, awe, and the value of life’s diversity. *Bioethics* 27, 411–418. doi: 10.1111/bioe.12051

Boldt, J. (2013b). Life as a technological product: philosophical and ethical aspects of synthetic biology. *Biol Theory* 8, 391–401. doi: 10.1007/s13752-013-0138-7

Boldt, J. (2018). Machine metaphors and ethics in synthetic biology. *Life Sci Soc Policy* 14. doi: 10.1186/s40504-018-0077-y

Braun, M., Ried, J., Dabrock, P., and Braun, M. (2013). From homo faber to homo creator? A theological-ethical expedition into the anthropological depths of synthetic biology. *Worldviews: Environment, Culture, Religion* 17, 36–47. doi: 10.1163/15685357-01701004

Chan, S. (2018). Research Translation and Emerging Health Technologies: Synthetic Biology and Beyond. Health Care Analysis 26, 310–325. doi: 10.1007/s10728-016-0334-2

Charpa, U. (2012). Synthetic biology and the Golem of Prague: Philosophical reflections on a suggestive metaphor. Perspect Biol Med 55, 554–570. doi: 10.1353/pbm.2012.0036

Chen, Y., Yin, Z., Shao, Z., and Xie, Q. (2015). The defence of artificial life by synthetic biology from ethical and social aspects. Journal of the College of Physicians and Surgeons Pakistan 25, 519–524.

Coyne, L. (2020). The ethics and ontology of synthetic biology: a neo-Aristotelian perspective. *Nanoethics* 14, 43–55. doi: 10.1007/s11569-019-00347-2

Dabrock, P. (2009). Playing god? synthetic biology as a theological and ethical challenge. *Syst Synth* *Biol* 3, 47–54. doi: 10.1007/s11693-009-9028-5

Deplazes, A., and Huppenbauer, M. (2009). Synthetic organisms and living machines: Positioning the products of synthetic biology at the borderline between living and non-living matter. *Syst* *Synth Biol* 3, 55–63. doi: 10.1007/s11693-009-9029-4

Douglas, T., Powell, R., and Savulescu, J. (2013). Is the creation of artificial life morally significant? *Studies in History and Philosophy of Science Part C: Studies in History and Philosophy of Biological and Biomedical Sciences* 44, 688–696. doi: 10.1016/j.shpsc.2013.05.016

Douglas, T., and Savulescu, J. (2010). Synthetic biology and the ethics of knowledge. *J Med Ethics* 36, 687–693. doi: 10.1136/jme.2010.038232

Funk, M., Steizinger, J., Falkner, D., and Eichinger, T. (2019). From Buzz to Burst—Critical Remarks on the Term ‘Life’ and Its Ethical Implications in Synthetic Biology. *Nanoethics* 13, 173–198. doi: 10.1007/s11569-019-00361-4

Glick, S. (2012). Synthetic biology: A Jewish view. *Perspect Biol Med* 55, 571–580. doi: 10.1353/pbm.2012.0039

Gómez-Tatay, L., Hernández-Andreu, J. M., and Aznar, J. (2016). A Personalist Ontological Approach to Synthetic Biology. *Bioethics* 30, 397–406. doi: 10.1111/bioe.12230

Gómez-Tatay, L., Hernández-Andreu, J. M., and Aznar, J. (2019). The Conception of Synthetic Entities from a Personalist Perspective. *Sci Eng Ethics* 25, 97–111. doi: 10.1007/s11948-017-9994-z

Gregorowius, D., and Deplazes-Zemp, A. (2016). Societal impact of synthetic biology: Responsible research and innovation (RRI). *Essays Biochem* 60, 371–379. doi: 10.1042/EBC20160039

Hagen, K. (2016). Science Policy and Concomitant Research in Synthetic Biology—Some Critical Thoughts. *Nanoethics* 10, 201–213. doi: 10.1007/s11569-016-0267-0

Häyry, M. (2017). Synthetic biology and ethics: Past, present, and future. *Cambridge Quarterly of Healthcare Ethics* 26, 186–205. doi: 10.1017/S0963180116000803

Heavey, P. (2013). Synthetic biology ethics: A deontological assessment. *Bioethics* 27, 442–452. doi: 10.1111/bioe.12052

Heavey, P. (2017). Consequentialism and the synthetic biology problem. *Cambridge Quarterly of Healthcare Ethics* 26, 206–229. doi: 10.1017/S0963180116000815

Heidari Feidt, R., Ienca, M., Elger, B. S., and Folcher, M. (2019). Synthetic Biology and the Translational Imperative. *Sci Eng Ethics* 25, 33–52. doi: 10.1007/s11948-017-0011-3

Heyd, D. (2012). Is there anything unique in the ethics of synthetic biology? *Perspect Biol Med* 55, 581–589. doi: 10.1353/pbm.2012.0041

Holm, S. (2012). Biological interests, normative functions, and synthetic biology. *Philos Technol* 25, 525–541. doi: 10.1007/s13347-012-0075-6

Holm, S. (2013). Organism and artifact: Proper functions in Paley organisms. *Studies in History and Philosophy of Science Part C: Studies in History and Philosophy of Biological and Biomedical Sciences* 44, 706–713. doi: 10.1016/j.shpsc.2013.05.018

Holm, S. (2017). The bioethicist who cried “synthetic biology” An analysis of the function of bioterrorism predictions in bioethics. *Cambridge Quarterly of Healthcare Ethics* 26, 230–238. doi: 10.1017/S0963180116000827

Holm, S. (2019). Deciding in the Dark: The Precautionary Principle and the Regulation of Synthetic Biology. *Ethics Policy Environ* 22, 61–71. doi: 10.1080/21550085.2019.1581419

Kaebnick, G. E., Gusmano, M. K., and Murray, T. H. (2014). The ethics of synthetic biology: next steps and prior questions. *Hastings Cent Rep* 44, S4–S26. doi: 10.1002/hast.392

Kelle, A. (2013). Beyond patchwork precaution in the dual-use governance of synthetic biology. *Sci Eng Ethics* 19, 1121–1139. doi: 10.1007/s11948-012-9365-8

Kotzé, M. (2020). Wicked solutions to wicked problems? A Christian ethical reflection on synthetic biology as nature conservation. *Philosophia Reformata* 85, 181–197. doi: 10.1163/23528230-8502A005

Laird, S. A., and Wynberg, R. P. (2016). Locating Responsible Research and Innovation Within Access and Benefit Sharing Spaces of the Convention on Biological Diversity: The Challenge of Emerging Technologies. *Nanoethics* 10, 189–200. doi: 10.1007/s11569-016-0268-z

Link, H. J. (2013). Playing God and the Intrinsic Value of Life: Moral Problems for Synthetic Biology? *Sci Eng Ethics* 19, 435–448. doi: 10.1007/s11948-012-9353-z

Macnaghten, P., Owen, R., and Jackson, R. (2016). Synthetic biology and the prospects for responsible innovation. *Essays Biochem* 60, 347–355. doi: 10.1042/EBC20160048

Magnin, T. (2015). Vulnerability at the Heart of the Ethical Implications of New Biotechnologies. *Human and Social Studies* 4, 13–25. doi: 10.1515/hssr

Mori, Y., Yoshizawa, G., Mori, Y., and Yoshizawa, G. (2011). Current situation of synthetic biology in Japan. *Journal of Disaster Research* 6, 476–481.

Newson, A. J. (2011). Current ethical issues in synthetic biology: Where should we go from here? *Account Res* 18, 181–193. doi: 10.1080/08989621.2011.575035

Newson, A. J. (2015). Synthetic biology: ethics, exceptionalism and expectations. *Macquarie Law Journal* 15, 45–58.

Pang, S., Lee, S. Y., and Seul, J. Y. (2017). Policy Challenges and Ethical Issues with the Breakthrough Technology: The Case of Synthetic Biology. *Science, Technology and Society* 22, 455–472. doi: 10.1177/0971721817723388

Preston, C. J. (2008). Synthetic biology: Drawing a line in Darwin’s sand. *Environ Values* 17, 23–39. doi: 10.3197/096327108X271932

Race, M. S., Moses, J., McKay, C., and Venkateswaran, K. J. (2012). Synthetic biology in space: Considering the broad societal and ethical implications. *Int J Astrobiol* 11, 133–139. doi: 10.1017/S1473550412000018

Rager-Zisman, B. (2012). Ethical and regulatory challenges posed by synthetic biology. *Perspect Biol Med* 55, 590–607. doi: 10.1353/pbm.2012.0043

Raho, J. A. (2014). Rethinking Nature Through Synthetic Biology. *Teoria-Rivista di Filosofia* 34, 93–111.

Rohregger, R., Sganzerla, A., and Simão-Silva, D. P. (2020). Synthetic biology and genetic manipulation: Risks, promises and responsibilities. *Ambiente e Sociedade* 23. doi: 10.1590/1809-4422ASOC20180196R3VU2020L4AO

Sandler, R. (2020). The ethics of genetic engineering and gene drives in conservation. *Conservation Biology* 34, 378–385. doi: 10.1111/cobi.13407

Saukshmya, T., and Chugh, A. (2010). Commercializing synthetic biology: Socio-ethical concerns and challenges under intellectual property regime. *J Commer Biotechnol* 16, 135–158. doi: 10.1057/jcb.2009.28

Schmidt, J. C. (2016). Prospective Technology Assessment of Synthetic Biology: Fundamental and Propaedeutic Reflections in Order to Enable an Early Assessment. *Sci Eng Ethics* 22, 1151–1170. doi: 10.1007/s11948-015-9673-x

Smith, K. (2013). Synthetic Biology: A Utilitarian Perspective. *Bioethics* 27, 453–463. doi: 10.1111/bioe.12050

Stirling, A., and Coburn, J. (2018). From CBA to Precautionary Appraisal: Practical Responses to Intractable Problems. *Hastings Center Report* 48, S78–S87. doi: 10.1002/hast.823

Takala, T. (2017). Finding hope in synthetic biology. *Cambridge Quarterly of Healthcare Ethics* 26, 239–245. doi: 10.1017/S0963180116000839

Thompson, P. B. (2012). Synthetic biology needs a synthetic bioethics. *Ethics Policy Environ* 15, 1–20. doi: 10.1080/21550085.2012.672676

Vallero, D. A., and Gunsch, C. K. (2020). Applications and Implications of Emerging Biotechnologies in Environmental Engineering. *Journal of Environmental Engineering* 146. doi: 10.1061/(asce)ee.1943-7870.0001676

van de Poel, I., and Robaey, Z. (2017). Safe-by-Design: from Safety to Responsibility. *Nanoethics* 11, 297–306. doi: 10.1007/s11569-017-0301-x

Wareham, C., and Nardini, C. (2015). Policy on synthetic biology: Deliberation, probability, and the precautionary paradox. *Bioethics* 29, 118–125. doi: 10.1111/bioe.12068
